# Genome-Wide Sensitivity Analysis of the Microsymbiont *Sinorhizobium meliloti* to Symbiotically Important, Defensin-Like Host Peptides

**DOI:** 10.1128/mBio.01060-17

**Published:** 2017-08-01

**Authors:** Markus F. F. Arnold, Mohammed Shabab, Jon Penterman, Kevin L. Boehme, Joel S. Griffitts, Graham C. Walker

**Affiliations:** a Department of Biology, Massachusetts Institute of Technology, Cambridge, Massachusetts, USA; b Department of Microbiology and Molecular Biology, Brigham Young University, Provo, UT, USA; Massachusetts General Hospital

**Keywords:** antimicrobial peptides, host-microbe interactions, symbiosis

## Abstract

The model legume species *Medicago truncatula* expresses more than 700 nodule-specific cysteine-rich (NCR) signaling peptides that mediate the differentiation of *Sinorhizobium meliloti* bacteria into nitrogen-fixing bacteroids. NCR peptides are essential for a successful symbiosis in legume plants of the inverted-repeat-lacking clade (IRLC) and show similarity to mammalian defensins. In addition to signaling functions, many NCR peptides exhibit antimicrobial activity *in vitro* and *in vivo*. Bacterial resistance to these antimicrobial activities is likely to be important for symbiosis. However, the mechanisms used by *S. meliloti* to resist antimicrobial activity of plant peptides are poorly understood. To address this, we applied a global genetic approach using transposon mutagenesis followed by high-throughput sequencing (Tn-seq) to identify *S. meliloti* genes and pathways that increase or decrease bacterial competitiveness during exposure to the well-studied cationic NCR247 peptide and also to the unrelated model antimicrobial peptide polymyxin B. We identified 78 genes and several diverse pathways whose interruption alters *S. meliloti* resistance to NCR247. These genes encode the following: (i) cell envelope polysaccharide biosynthesis and modification proteins, (ii) inner and outer membrane proteins, (iii) peptidoglycan (PG) effector proteins, and (iv) non-membrane-associated factors such as transcriptional regulators and ribosome-associated factors. We describe a previously uncharacterized yet highly conserved peptidase, which protects *S. meliloti* from NCR247 and increases competitiveness during symbiosis. Additionally, we highlight a considerable number of uncharacterized genes that provide the basis for future studies to investigate the molecular basis of symbiotic development as well as chronic pathogenic interactions.

## IMPORTANCE

Soil rhizobial bacteria enter into an ecologically and economically important symbiotic interaction with legumes, in which they differentiate into physiologically distinct bacteroids that provide essential ammonia to the plant in return for carbon sources. Plant signal peptides are essential and specific to achieve these physiological changes. These peptides show similarity to mammalian defensin peptides which are part of the first line of defense to control invading bacterial populations. A number of these legume peptides are indeed known to possess antimicrobial activity, and so far, only the bacterial BacA protein is known to protect rhizobial bacteria against their antimicrobial action. This study identified numerous additional bacterial factors that mediate protection and belong to diverse biological pathways. Our results significantly contribute to our understanding of the molecular roles of bacterial factors during legume symbioses and, second, provide insights into the mechanisms that pathogenic bacteria may use to resist the antimicrobial effects of defensins during infections.

## INTRODUCTION

Leguminous plants establish a mutually beneficial symbiosis with soil bacteria, termed rhizobia, that enables them to acquire nitrogen in the form of ammonia (NH_3_). *Medicago sativa* (alfalfa) and *Medicago truncatula*, which are members of the inverted-repeat-lacking clade (IRLC) legumes, secrete flavonoids that induce the expression and secretion of lipo-oligosaccharide nodulation (Nod) factor by the soil bacterium *Sinorhizobium meliloti* (1). Nod factor induces root hair curling to entrap nearby bacteria and elicits the formation of the nodule primordium within the root cortex. Entrapped *S. meliloti* bacteria divide along preformed tubular structures, termed infection threads (ITs), until they reach the nodule primordium cells where they are endocytosed into the cytoplasm of specialized nodule cells. The membrane-bound compartments within these cells that enclose the invading bacteria are termed symbiosomes (1). Inside the symbiosomes, *S. meliloti* bacteria differentiate into elongated bacteroids which, when mature, express nitrogenase to fix atmospheric nitrogen (N_2_) into NH_3_ for the plant host. In return, symbiotic bacteria obtain fixed carbon in the form of dicarboxylic acids from the plant (1).

The differentiation of free-living *S. meliloti* bacteria into bacteroids is mediated by a cocktail of more than 700 nodule-specific cysteine-rich (NCR) signaling peptides that are exclusively expressed in the newly formed root nodules (2, 3). NCR peptide expression is tightly regulated during the different phases of bacterial nodule invasion, and infecting bacteria encounter these peptides in successive waves (4). NCR peptides have conserved motifs of either four or six cysteines in common but otherwise show a high degree of sequence and charge diversity. They are structurally very similar to characterized defensins, and they are often referred to as defensin-like peptides (2, 5). NCR peptides are delivered by a dedicated secretory apparatus and then drive the symbiotic differentiation of the intracellular bacteria into bacteroids that is essential for nitrogen fixation (3, 6). Underscoring the physiological importance of plant NCR peptides, disruption of the genes encoding either NCR211 (7) or NCR169 (8) was recently shown to result in symbiotic deficiency *in planta*. It was also shown that the cysteine residues of NCR169 are critical for its symbiotic functions (8).

An extensively studied member of the NCR peptide family is NCR247, a cationic 24-amino-acid peptide with four cysteines that presumably form intramolecular bridges in the oxidizing environment of the secretory system. At relatively low concentrations, the NCR247 peptide has been shown to have significant effects on the *S. meliloti* transcriptome *in vitro* (*ex planta*) and induces bacteroid-like features, such as a block in cell division, an increase in cell size, and endoreduplication (3, 9). Paradoxically, at higher doses, NCR247 possesses antimicrobial activity toward free-living *S. meliloti* (3, 9–11). Intramolecular disulfide cross-linking of NCR247 into the three possible regio-isomers has recently been reported to strikingly alter the effects of the peptide on *S. meliloti in vitro* (12). NCR247 also has potent antimicrobial activity against clinically relevant microbes (11) including *Candida albicans* (13).

The *S. meliloti bacA* gene, long recognized as a critical symbiotic factor (14) provides protection from the NCR247 peptide *in vitro* (10). Without *bacA*, *S. meliloti* cells typically lyse within nascent symbiosomes, though this lysis phenotype is suppressed in a host secretory mutant that is unable to deliver NCR peptides to symbiosomes (10). Thus, the antimicrobial activity of NCR peptides has relevance *in planta*. Together, these observations suggest that *S. meliloti* must rely on mechanisms that establish basal resistance to the antimicrobial action of NCR peptides to be symbiotically proficient.

In this study, we used *mariner* transposon mutagenesis followed by high-throughput sequencing of all transposon insertion sites (Tn-seq) to obtain a global view of the genes that protect or sensitize *S. meliloti* cells when they are exposed to the NCR247 peptide (15). To provide a point of comparison, we also used the well-characterized, unrelated cationic peptide polymyxin B (PMB), which is a nonribosomally synthesized amphipathic antimicrobial compound that primarily targets the cell envelopes of Gram-negative bacteria (16, 17).

Until now, only the *bacA* gene was known to provide resistance against the antimicrobial effects of the NCR247 peptide (10). We identified 77 other genes that affect the relative competitiveness of *S. meliloti* during NCR247 exposure, either necessary for resistance or necessary for sensitivity, and 54 that affected the competitiveness of *S. meliloti* during PMB challenge. Our results suggest that *S. meliloti* possesses a seemingly broad and diverse array of mechanisms to modulate the effects of NCR247 exposure to either be beneficial (signaling for symbiosis) or detrimental (antimicrobial effects) *in vitro*. To our knowledge, there is only one study that used a transposon-based screen, which was performed on human gut commensals testing for their sensitivity to the non-host antimicrobial peptide (AMP) PMB (18). Our results open the door to further molecular-level investigations into symbiotic compatibility and into bacterial responses to antimicrobial peptides more generally. To our knowledge, no Tn-seq study has been published to identify bacterial factors that protect or sensitize host-invading bacteria to the antimicrobial activity of host innate antimicrobial peptides in any host-microbe-related system.

## RESULTS

### Using Tn-seq to identify genes affecting competitiveness during stress caused by NCR247 or PMB.

**FIG 1:**
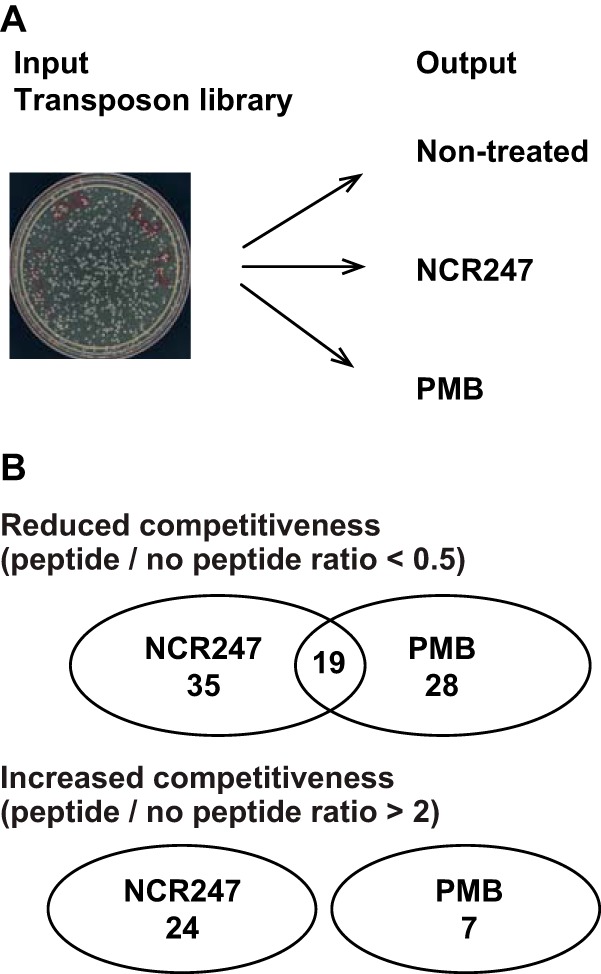
Overview of Tn-seq experiments. (A) Schematic overview of the experimental design indicating the input and output samples that were generated for sequencing. (B) Overview of the results obtained. The numbers of genes found to decrease competitiveness and genes found to increase bacterial competitiveness are shown with the indicated cutoff values and the number of genes with overlapping functions that affect NCR247 and PMB competitiveness.

In this study, we utilized *mariner* transposon mutagenesis followed by high-throughput sequencing of all transposon insertion sites (Tn-seq) to identify genes that affect *S. meliloti* competitiveness during exposure to the NCR247 peptide and polymyxin B (PMB). We first created a library of approximately 159,000 *mariner* transposon insertions within all annotated open reading frames of *S. meliloti* resulting in an average insertion density of approximately 23 insertions per 1,000 bp (19, 20). A summary of sequencing statistics can be found in Table 1, including total read counts that were observed and normalization factors for each sample. We then exposed cultures of the transposon library to either the NCR247 peptide or PMB as described in Materials and Methods before sequencing of insertion sites (Fig. 1A). The nature of the transposon with its flanking MmeI restriction sites allowed us to uniquely map the majority of transposon insertions to annotated genes in the library by sequencing the genomic DNA (gDNA) (16 bp) adjacent to each transposon as previously described (19, 21). Each gene that is discussed in this study was found to contain multiple transposon insertions throughout their open reading frames (for examples, see Fig. S1A in the supplemental material). Because bacteria in symbiosomes are likely exposed to NCR peptides in their oxidized form (22) and the exact disulfide connectivity for any NCR peptide *in planta* is not known but can influence biological action (12), we used a mixture of all three possible disulfide-cross-linked NCR247 regio-isomers (12). To provide a comparison with an intensively studied antimicrobial peptide, we carried out a parallel experiment in which we used PMB. Like NCR247 (net 6 positive charges at pH 7.0 [12, 23]), PMB (net 5 positive charges at pH 7.0 [24]) is cationic but structurally different compared to NCR247. PMB can kill either by disruption of the inner membranes of Gram-negative bacteria or by the production of hydroxyl radicals that eventually result in the inactivation of Fe-S clusters and subsequent cell death by DNA, lipid, or protein damage (for an overview, see reference 17).

10.1128/mBio.01060-17.1 FIG S1  Example of output data from Tn-seq experiment. (A) Tn-seq results for three representative genes. The left panels cover the complete length of each gene indicating the locations with transposon insertions. All experimental conditions are depicted. The data range indicates the height of the *y* axis. In the middle panels, the average read count numbers per transposon insertion for each experimental condition is shown. The right panels show the computed data as used for the interpretation of the data depicting the calculated competition indexes. (B) Tn-seq results for gene *smc03872*. The top panel covers the complete length of the gene indicating the locations with transposon insertions. All experimental conditions are depicted. The data range indicates the height of the *y* axis. The bottom left panel shows the average read count numbers per transposon insertion for each experimental condition. The bottom right panel shows the computed data as used for the interpretation of the data depicting the calculated competition indexes. Download FIG S1, PDF file, 0.8 MB. Copyright © 2017 Arnold et al. 2017 Arnold et al. This content is distributed under the terms of the Creative Commons Attribution 4.0 International license.

**TABLE 1:** Tn-seq analysis statistics^a^

Biological replicate and sample	Total no. of reads	% reads with no Tn sequence	No. of reads too short	No. of reads used for analysis	AR^b^ (%)	NF^c^	Total no. of normalized reads	No. of reads/Tn site
Biological replicate 1								
Input	21,652,251	5.20	36,631	20,489,022	80.40	0.844	18,274,500	129
Nontreated	20,304,098	6.94	29,769	18,865,247	80.09	0.920	18,679,770	119
NCR247 treated	20,121,669	8.14	34,245	18,450,409	80.80	0.921	18,532,057	116
PMB treated	18,401,067	7.18	30,931	17,049,767	80.66	0.998	18,364,265	107
Biological replicate 2								
Input	20,153,000	12.72	30,042	17,560,139	81.50	0.943	19,004,279	111
Nontreated	22,731,094	7.97	33,510	20,885,738	80.66	0.811	18,434,917	132
NCR247 treated	18,798,923	7.77	30,051	17,307,732	80.11	1.000	18,798,923	109
PMB treated	18,906,038	8.75	28,138	17,224,367	79.88	0.994	18,792,602	109

a Tn, transposon.

b AR, alignment rate.

c NF, normalization factor as determined by normalizing all samples to the sample with the lowest number of intergenic reads.

Under conservative data analysis conditions (described in Materials and Methods), 5,386 out of the 6,218 annotated *S. meliloti* genes (25) had substantial numbers of transposon insertions ranging from 100 to 27,027 normalized read counts per gene in the input transposon library, and these insertions did not substantially impair bacterial fitness in the absence of antimicrobial peptides (Fig. 1A and Fig. S1A). A total of 832 genes were not further analyzed (Table S1). Of these 832 genes, 741 contained either 100 or fewer read counts per gene in either of the two biological replicates. The remaining 91 genes were excluded because transposon insertions in these genes significantly impaired bacterial growth even without NCR247 or PMB stress during our experiments, which were carried out in 3-(*N*-morpholino)propanesulfonic acid (MOPS) buffer supplemented with Casamino Acids (described in Materials and Methods). The 832 excluded genes were therefore considered essential, pseudoessential, or otherwise recalcitrant to transposon insertions due to their small size or to a lack of TA sites required for *mariner* transposon targeting.

10.1128/mBio.01060-17.6 TABLE S1  Essential and underrepresented genes. Download TABLE S1, XLSX file, 0.1 MB. Copyright © 2017 Arnold et al. 2017 Arnold et al. This content is distributed under the terms of the Creative Commons Attribution 4.0 International license.

To identify genes affecting *S. meliloti* competitiveness, the normalized total transposon read counts per gene for each peptide treatment condition were compared to counts from no-peptide control treatments (Fig. 1A). To achieve this, the number of total read counts per gene of either the NCR247 peptide or PMB were divided by the total read counts per gene of the untreated cell population after outgrowth. The resulting competition index (CI) (treated/untreated) was used throughout this study to compare the competitiveness of the treated transposon mutants, whereby a CI of 1 represents no change of transposon insertion-induced competitiveness. If the relative representation of a transposon insertion in a treated culture was changed twofold or more in either direction in both independent biological replicates, genes were classified to be important for the competitiveness of *S. meliloti* during treatment with either NCR247 or PMB. Thus, a CI value smaller than 0.5 suggests that the given gene provides a competitive advantage, and a CI value larger than 2 suggests that a gene provides a competitive disadvantage (Fig. S1A).

We identified a total of 54 genes (NCR247 challenge) and 47 genes (PMB challenge) which, when mutated, increase peptide sensitivity (Fig. 1B). Nineteen of these genes provide a competitive advantage during treatment with either the NCR247 peptide or PMB, suggesting that they participate in pathways that provide general protection from cationic antimicrobial peptides. Additionally, we found 24 genes (NCR247 challenge) and 7 genes (PMB challenge) which, when mutated, decrease peptide sensitivity (Fig. 1B). Genes in this category were unique to each treatment. Genes influencing NCR247 or PMB sensitivity or resistance are involved in various pathways affecting cell envelope structure and stability, while others encode transcriptional regulators, ribosome-associated proteins, and various as-yet uncharacterized proteins. We have classified most of these genes into three major groups that are highlighted and discussed in the sections below. Comprehensive lists of all the genes identified as affecting competitiveness during NCR247 and PMB treatment are listed in the supplemental material (Tables S2 and S3). Complete analysis data files can be found deposited online (https://doi.org/10.5061/dryad.32152).

10.1128/mBio.01060-17.7 TABLE S2  Genes with altered competitiveness caused by transposon insertions during NCR247 exposure. Download TABLE S2, XLSX file, 0.03 MB. Copyright © 2017 Arnold et al. 2017 Arnold et al. This content is distributed under the terms of the Creative Commons Attribution 4.0 International license.

10.1128/mBio.01060-17.8 TABLE S3  Genes with altered competitiveness caused by transposon insertions during PMB exposure. Download TABLE S3, XLSX file, 0.02 MB. Copyright © 2017 Arnold et al. 2017 Arnold et al. This content is distributed under the terms of the Creative Commons Attribution 4.0 International license.

### Cell envelope-associated polysaccharides affect the competitiveness of *S. meliloti* during NCR247 and PMB exposure. (i) Lipopolysaccharides.

**FIG 2:**
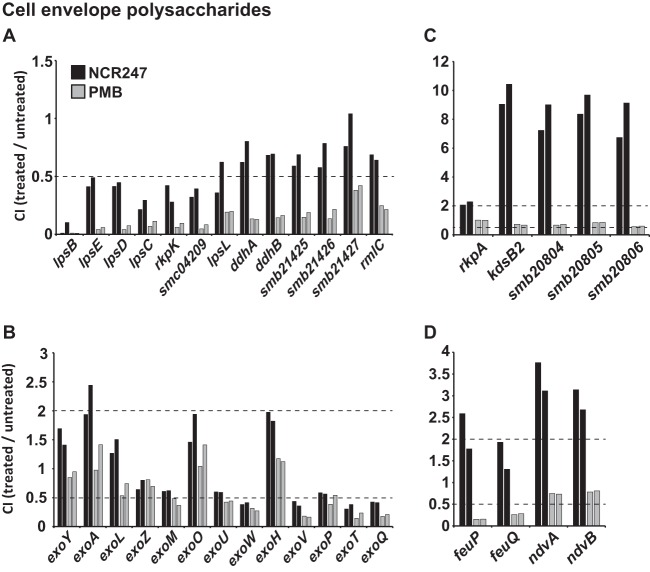
*S. meliloti* cell envelope polysaccharides affect NCR247 competitiveness differentially. (A) Competition index (CI) values for known LPS biosynthesis genes with competition disadvantage for PMB, (B) genes involved in succinoglycan biosynthesis in the order they act in the pathway, (C) KPS genes identified to affect NCR247 competitiveness, and (D) genes known to affect cyclic β-glucan biosynthesis and export are shown. The results for NCR247 (black bars) and PMB (gray bars) are shown for both biological replicates for each condition. The dashed lines indicate the cutoff CI values of ≤0.5 and ≥2, representing reduced and increased competitiveness, respectively.

Lipopolysaccharide (LPS) is the outermost layer of the Gram-negative cell envelope, and our results suggest that it plays an important role in protecting *S. meliloti* against the antimicrobial activities of NCR247 and PMB. We found that transposon insertions in genes that affect LPS structure in *S. meliloti* increase bacterial sensitivity during NCR247 or PMB treatment (CIs of <0.5) (Fig. 2A). Among the genes that provide protection against NCR247 and PMB is the *lpsBCDE* cluster whose products have been shown to modify the LPS core sugars in *S. meliloti* (26). We also found that genes *rkpK* and *smc04209* whose products are known to affect LPS structure provided protection against the NCR247 peptide, in addition to PMB (Fig. 2A) (26). Mutation of these genes previously has been shown to adversely affect the *S. meliloti-M. truncatula* symbiosis and to increase sensitivity to PMB, mellitin, and poly-l-lysine which is reproduced in our results for PMB (Fig. 2A) (26–28). Our identification of these genes therefore serves to validate the results that we present in this study (Fig. 2A). Also, it has been shown previously that *Escherichia coli* LPS core biosynthesis enzymes are involved in protection from a human defensin peptide, HD5 (29). To confirm that changes to LPS affect NCR247 sensitivity, we made use of a readily available *lpsB*::Tn*phoA* mutant (28), and we were able to show that this strain is more sensitive to NCR247 than a wild-type *S. meliloti* strain (Fig. S2A).

10.1128/mBio.01060-17.2 FIG S2  Existing *S. meliloti* mutant strains display altered sensitivity to NCR247 compared to a wild-type strain. (A and B) The indicated strains were treated with 6 µM NCR247-AR for the indicated length of time in MOPS-GS Cas buffer and recovered in triplicates. (C) *feuP* mutant bacteria were coinoculated with *S. meliloti* wild-type bacteria starting at a ratio of 1% to 99% (mutant/wild type). Untreated and NCR247-AR-treated cultures were recovered at the defined time points, and then the percentage of *feuP* mutant bacteria at any given time point was plotted. All data shown are representative of at least two independent experiments. Download FIG S2, PDF file, 0.8 MB. Copyright © 2017 Arnold et al. 2017 Arnold et al. This content is distributed under the terms of the Creative Commons Attribution 4.0 International license.

Furthermore, we found that mutations in seven additional genes (*lpsL*, *ddhA*, *ddhB*, *smb21425*, *smb21426*, *smb21427*, and *rmlC* [*smc21428*]) resulted in only moderately decreased competitiveness during NCR247 exposure (CI values just above 0.5) but significantly decreased competitiveness during PMB exposure (CI of <0.5) (Fig. 2A). Mutations in genes *lpsL*, *ddhB*, and *smb21425* affect bacterial LPS structure and have been shown to play important roles in symbiosis (26). An *S. meliloti rmlC* mutant has not yet been tested for changes in LPS, but it has been suggested that *rmlC* is part of a gene cluster involved in surface carbohydrate biosynthesis or assembly (30). *ddhA*, *smb21426*, and *smb21427* mutants have not been studied before regarding their LPS structure or PMB sensitivity, but the genes most likely encode proteins that are involved in pathways related to the products of genes *ddhB* and *smb21425*, respectively, as suggested by their presence in common transcriptional units.

### (ii) Extracellular polysaccharides: succinoglycan and capsular polysaccharide.

Succinoglycan is a secreted bacterial polysaccharide essential for the formation and directed growth of plant root-hair infection threads (1, 31, 32). Our results suggest that *S. meliloti* succinoglycan exerts protective functions against the antimicrobial activities of the NCR247 peptide and PMB. Mutations in the succinoglycan biosynthetic genes *exoW*, *exoV*, *exoT*, and *exoQ* significantly decreased competitiveness during NCR247 and PMB exposure, while mutations in two additional genes (*exoM* and *exoU*) significantly reduced competitiveness only during PMB exposure (Fig. 2B). Interestingly, the four *exo* genes that affect competitiveness during both NCR247 and PMB antimicrobial stress coordinate the final steps of succinoglycan processing. These steps are the addition of a pyruvyl-modifying group to the final glucose residue (*exoV*) and the export and polymerization of the succinoglycan monomers into low-molecular-weight (LMW) (*exoT*) or high-molecular-weight (HMW) (*exoQ*) polymers (31). The poor competitiveness of mutants lacking functional ExoP, which participates in the formation of both HMW and LMW succinoglycan, was quite similar to that of the *exoQ*, *exoV*, *exoT*, and *exoW* mutants, but we had not scored it as significant in our analysis, as its CI values were just above 0.5 (Fig. 2B). Testing the two existing *exoV2* and *exoV92* (33) mutants showed that they are significantly more sensitive to the antimicrobial activity of NCR247 than an *S. meliloti* wild-type strain under conditions comparable to those of our Tn-seq experiment (Fig. S2B). In contrast to the *lpsB*::Tn*phoA* mutant that we tested (one NCR247 treatment over a period of 10 h), the two tested *exoV* mutants required two treatments with NCR247 over a period of 24 h to observe a difference in sensitivity.

In addition to succinoglycan, *S. meliloti* produces another extracellular polysaccharide, a capsular polysaccharide often referred to as K-antigen polysaccharide or KPS, consisting of a homopolymer of β-(2-7)-linked 3-deoxy-d-manno-oct-2-ulopyranosonic acid (Kdo) attached to a lipid anchor that can contribute to symbiosis (34–36). Our data suggest that *S. meliloti* 1021 KPS affects NCR247 sensitivity. However, the observed results were different in two respects from the effects of mutating genes involved in LPS or succinoglycan biosynthesis. First, mutating genes that affect *S. meliloti* 1021 KPS (*rkpA*, *kdsB2*, *smb20804*, and *smb20805* [37]) resulted in a significant increase of the CI in the presence of the NCR247 peptide, suggesting that KPS has a sensitizing function toward NCR247 in *S. meliloti* 1021. Second, we observed that mutations in genes that affect KPS alter the competitiveness only during NCR247 treatment but not in the presence of PMB (Fig. 2C).

### (iii) Cyclic β-glucans.

Cyclic β-glucans (CβGs) are modified, circular polysaccharides composed of 17 to 40 glucose residues that are essential for the early stages of the *S. meliloti-Medicago* symbiosis (38, 39). Our results imply that, just like KPS, CβGs play a sensitizing role during NCR247 treatment (Fig. 2D). Their export into the periplasm via the ATP-dependent exporter NdvA is regulated by the two-component system FeuQP after their synthesis by the cytoplasmic CβG synthase, NdvB (40, 41). Transposon insertions in the genes encoding NdvA and NdvB significantly increased the competitiveness of *S. meliloti* 1021 in the presence of the NCR247 peptide (Fig. 2D). Using a *feuP* deletion mutant, we could confirm that this strain shows increased resistance to NCR247 compared to a wild-type strain in a competition viability assay (Fig. S2C). Transposon insertions in *feuQ* or *feuP* had the opposite effect during PMB stress by decreasing the competitiveness, but mutating the *ndvA* and *ndvB* genes showed little to no effect (Fig. 2D).

### Cell envelope modifications and membrane proteins. (i) Very-long-chain fatty acids.

**FIG 3:**
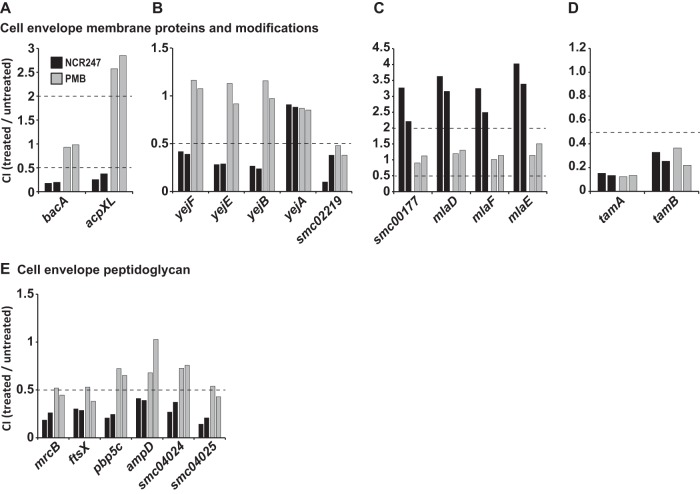
*S. meliloti* cell envelope membrane proteins and peptidoglycan can modulate NCR247 competitiveness. (A) Competition index (CI) values for genes that are known to affect very-long-chain fatty acid (VLCFA) modifications in *S. meliloti*. (B) CI values for single components of a putative microcin C ABC transporter. (C) CI values for single components of a putative ABC transporter involved in outer membrane lipid asymmetry. (D) CI values for single components of two genes encoding two putative components of an *S. meliloti* translocation and assembly (Tam) system. (E) CI values for genes encoding putative cell wall peptidoglycan-modifying enzymes. The results for NCR247 (black bars) and PMB (gray bars) are shown for both biological replicates for each condition. The dashed lines indicate the cutoff CI values of ≤0.5 and ≥2, representing reduced and increased competitiveness, respectively.

Transposon insertions in gene *acpXL*, which encodes an acyl carrier protein that is part of the gene cluster responsible for the biosynthesis and attachment of very-long-chain (C_28_ and C_30_) fatty acids (VLCFAs) to lipid A (42–44), resulted in significantly reduced competitiveness in the presence of the NCR247 peptide, suggesting a protective function against the antimicrobial activity of NCR247 (Fig. 3A). Mutation of the remaining genes of the cluster (*lpxXL*, *adhA2XL*, *fabF1XL*, *fabF2XL* and *fabZXL* [42] [also see Table S1]) caused a significant growth disadvantage in our MOPS growth medium, and so these genes do not qualify for our analysis under our conservative analytical criteria. It has been shown that these mutants require at least some salt in their growth medium (42). We used an existing *acpXL*::*pK18mobGII* insertion mutant (43, 45) to confirm that this mutant shows increased sensitivity toward NCR247 compared to the wild-type *S. meliloti* strain (Fig. S2A). In contrast to our findings during NCR247 exposure, we did discover that mutating the *acpXL* gene results in a significant increase in competitiveness in the presence of PMB (Fig. 3A). This observation provides more evidence for differences in the mechanisms controlling sensitivity to NCR247 and PMB in *S. meliloti*.

### (ii) Membrane proteins.

The BacA protein is a well-characterized membrane protein and has previously been shown to protect *S. meliloti* 1021 against the antimicrobial activity of the NCR247 peptide (10). As anticipated, the *bacA* gene was found to be important for NCR247 competitiveness as indicated by significantly reduced CI values resulting from transposon insertions in the *bacA* gene, an additional validation of our screen (Fig. 3A). The exact mode of action and function of BacA remain to be elucidated, but BacA has been shown to alter the transport of diverse peptide substrates across the inner membrane (46, 47), including NCR peptides (48), and also to affect the VLCFA content in the outer membrane of *S. meliloti* (49, 50). Furthermore, the identification of the well-studied *bacA* gene as a factor important for NCR247 sensitivity in our study is yet another finding that helps to validate the approach that we utilized.

Our study also identified a number of genes that are uncharacterized (Table S2 and S3). Using homology analyses and NCBI BLAST (http://blast.ncbi.nlm.nih.gov/Blast.cgi), we have assigned putative roles to select gene products and briefly speculate about their putative functions and their potential relationship to NCR247 competitiveness based on their CIs. A subset of these uncharacterized genes encode proteins that form two putative inner membrane ABC transport systems. The constituents of the first putative ABC transport system show homology to the *E. coli* YejABEF microcin C (McC) ABC importer (51) that has also been associated with resistance to antimicrobial peptides in two bacterial pathogens (52, 53). Transposon insertions in genes *smc02829* (*yejF*; cytoplasmic ATPase), *smc02830* and *smc02831* (*yejE* and *yejB*, respectively; permease domains), encoding the SMc028XX ABC system appear to reduce *S. meliloti* 1021 competitiveness as manifested by CI values of ≤0.5 after NCR247 exposure (Fig. 3B). Interestingly, mutations in an adjacent gene (*smc02832*; *yejA*) that encodes a periplasmic substrate binding protein did not affect the CI during NCR247 treatment (Fig. 3B). In the genome of *S. meliloti*, the putative *yejABEF* operon is reversed, beginning with *yejF* and ending with *yejA*. We found that *smc02219*, encoding a putative periplasmic binding protein whose inactivation did reduce competitiveness during NCR247 exposure shows similarity to YejA (28%) (Fig. 3B). Either the putative *S. meliloti* 1021 Yej transport system does not require a periplasmic binding protein to protect against NCR247 or an orphan periplasmic binding protein such as SMc02219 may substitute for the putative *S. meliloti* 1021 YejA protein (SMc02832) during NCR247 challenge to complete the SMc028XX ABC transporter to fulfill its protective function by forming a complete importer. However, uncovering the molecular functions of this ABC transport system will be one objective for future studies. Just like YejABEF for McC in *E. coli*, this transport system might be involved in transport of the NCR247 peptide. Components of the second ABC transport system show homology to the *E. coli* membrane lipid asymmetry (MLA) system. This system helps to maintain the lipid asymmetry in the outer membrane of the Gram-negative cell envelope (54). Mutating genes *smc00174* (*mlaE*; permease domain), *smc00175* (*mlaF*; cytoplasmic ATPase), *smc00176* (*mlaD*; periplasmic binding protein), and *smc00177* (auxiliary component lipoprotein), encoding the SMc0017X ABC system increases bacterial competitiveness during NCR247 challenge as indicated by significantly increased CI values (Fig. 3C). Assuming similar lipid partitioning activity in *S. meliloti* 1021, it is possible that altered outer membrane lipid composition in these mutants somehow protects cells from NCR247. It should be noted that the results obtained for the transposon insertions in the genes encoding these two ABC transport systems are specific to NCR247 and that these ABC transport systems do not affect bacterial competitiveness during PMB treatment.

Genes *smc03097* (putative *tamA*) and *smc03096* (putative *tamB*) encode proteins that show homology to a translocation and assembly module (Tam), and transposon insertions in these genes decrease the competitiveness during NCR247 and PMB exposure (Fig. 3D). Tam systems have been reported to play important roles in the insertion of β-barrel proteins into the cell envelopes of Gram-negative bacteria, and some Tam systems are known to be essential for bacterial virulence in their respective hosts (55).

### (iii) Peptidoglycan.

Transposon insertions in six genes encoding proteins that are putatively involved in peptidoglycan (PG) processing resulted in significantly reduced CI levels during NCR247 stress (Fig. 3E). The respective genes and their putative functional annotations include the two carboxypeptidases *smc00122* (*mrcB*) and *smc01188* (*pbp5C*), a cell division factor (*smc00716*, *ftsX*), an amidase (*smc01854*, *ampD*), a transglycosylase (*smc04024*), and a hydrolase (*smc04025*). While most of these genes are not characterized, *smc01188*, encoding a putative d-alanyl-d-alanine-carboxypeptidase is 49% similar to the *Bradyrhizobium* sp. strain ORS278 DD-carboxypeptidase BRADO4549. BRADO4549 has been found critical for the legume symbiosis of *Bradyrhizobium* bacteria with plants of *Aeschynomene* plant species which also produce NCR peptides (48, 56). Notably, although trending the same direction as during NCR247 exposure, mutations in these genes did not significantly reduce the CI in the presence of PMB in either direction (Fig. 3E).

### Non-cell envelope-associated proteins. (i) Transcriptional regulators.

**FIG 4:**
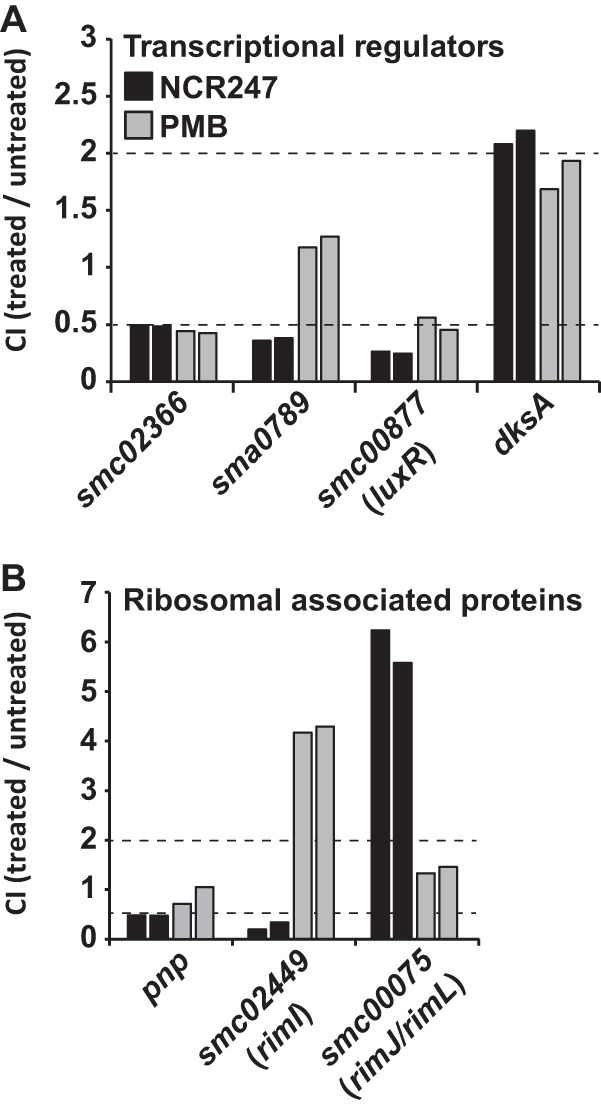
Genes that encode cytoplasmic effector proteins alter *S. meliloti* NCR247 competitiveness. (A) Competition index (CI) values for genes encoding putative transcriptional regulators. (B) Putative *S. meliloti* acetyltransferases and ribosome-associated proteins that affect NCR247 competitiveness. The results for NCR247 (black bars) and PMB (gray bars) are shown for both biological replicates for each condition. The dashed lines indicate the cutoff CI values of ≤0.5 and ≥2, representing reduced and increased competitiveness, respectively.

In addition to FeuP/FeuQ, we identified transcriptional regulators that significantly affect the CI during NCR247 challenge (Fig. 4A). These include the putative transcriptional regulator SMc02366 that shows 60% amino acid sequence similarity to FeuP and the putative histidine kinase SMc02367 that shows 48% amino acid sequence similarity to FeuQ (Fig. 4A); it seems likely that these are a two-component pair that controls a regulon yet to be determined. Furthermore, we found transposon insertions in gene *sma0789*, encoding a transcriptional regulator of the GntR family with unknown function that significantly decrease the competitiveness during NCR247 stress (Fig. 4A). Mutations in gene *smc00877*, which encodes a transcriptional regulator of the LuxR family, decrease the CI in the presence of the NCR247 peptide (57). Another transcriptional regulator whose loss of function significantly increases the CI for NCR247 was *dksA*, which was previously found to be an important regulator of the stringent response and critical for effective symbiosis of *S. meliloti* with the legume alfalfa (*M. sativa*) (58).

### (ii) Ribosome-associated proteins.

Interestingly, we also found ribosome-associated proteins to affect the competitiveness of *S. meliloti* during NCR247 treatment (Fig. 4B). Disrupting gene *smc02449*, encoding a putative ribosomal-protein-alanine *N*-acetyltransferase (probably RimI), significantly lowers *S. meliloti* competitiveness during NCR247 treatment but has the opposite effect under PMB stress (Fig. 4B). In contrast, disrupting gene *smc00075*, encoding a putative RimJ/RimL homologue significantly increases the competitiveness of *S. meliloti* under NCR247 stress (Fig. 4B). RimJ and RimL acetylate the N termini of ribosomal proteins S5 and L12, respectively (59, 60). Additionally, transposon insertions in the annotated polynucleotide phosphorylase (*pnp*) gene result in a significant decrease in CI during NCR247 challenge (Fig. 4B). Polynucleotide phosphorylase (PNPase) plays a major role in regulation of RNA levels in bacterial cells as well as participating in tRNA processing and 16S rRNA maturation (61, 62).

### The putative periplasmic peptidase SMc03872 mediates NCR247 resistance.

**FIG 5:**
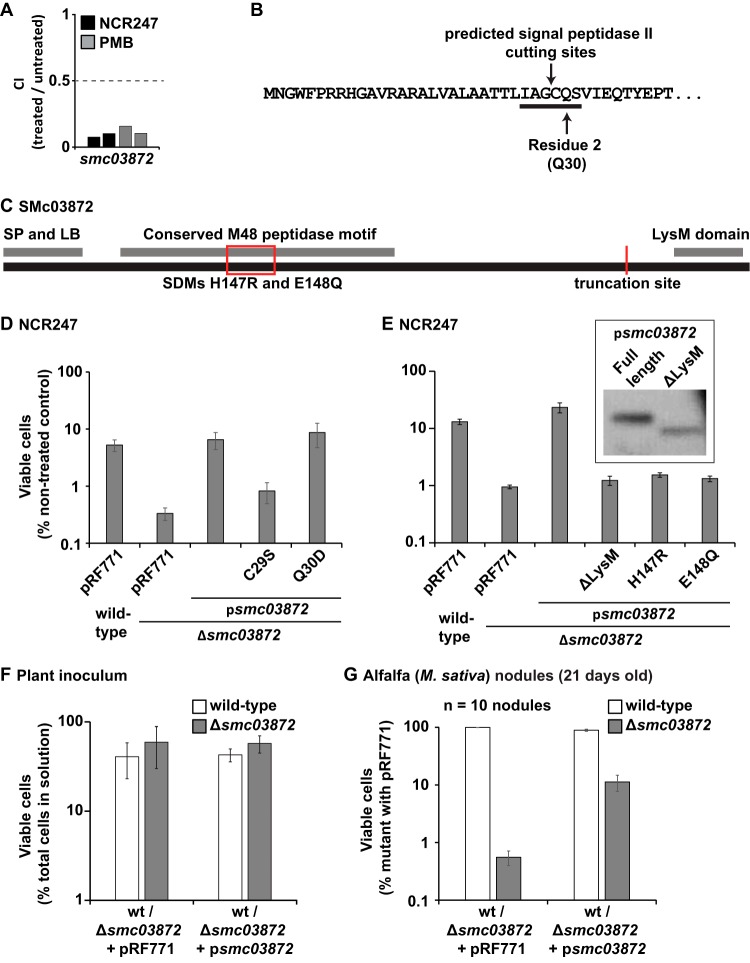
The putative *S. meliloti* metallopeptidase protects against NCR247 and provides a competitive disadvantage. (A) Tn-seq results for gene *smc03872* showing both replicates for NCR247 and PMB. (B) N-terminal amino acid sequence of the SMc03872 protein highlighting the conserved lipobox and predicted signal peptidase cutting site. (C) Schematic representation of SMc03872 highlighting the predicted N-terminal signal peptide (SP) with the conserved lipobox (LB), the conserved metallopeptidase domain, and the conserved C-terminal LysM domain. The approximate locations of the two introduced site-directed mutations and the truncation site are indicated in red. (D and E) Early-log-phase cells of the indicated wild-type and mutant *S. meliloti* strains were treated with two doses of 6 µM NCR247-AR over a time course of 24 h. (F) Bacterial composition of alfalfa plant inoculum of the indicated strains. Values are means ± standard deviations (error bars). wt, wild type. (G) Bacterial cells recovered from 21-day-old nodules represented by the fraction of bacteria recovered with a peptidase mutant with and without a complementation construct. Values are means ± standard errors (error bars) for 10 nodules. All experimental results shown are representative for trends observed in at least two independent experiments.

We studied the previously uncharacterized gene *smc03872* in greater depth because it is highly conserved in alphaproteobacteria and showed strong effects on the resistance of *S. meliloti* 1021 toward the NCR247 peptide and PMB (Fig. 5). Transposon insertions in gene *smc03872* significantly reduce the CI in the presence of NCR247 and PMB, indicating a protective function of the protein encoded by this gene (Fig. 5A and Fig. S1B). At its N terminus, the SMc03872 protein contains a predicted signal sequence, a lipobox with a glutamine at the residue 2 position, indicating that SMc03872 is an outer membrane lipoprotein, and a conserved M48 peptidase domain (Fig. 5B and C) (63). At its C terminus, SMc03872 possesses a LysM domain, which is known for its PG binding capabilities (Fig. 5C) (64). Therefore, the peptidase and LysM regions are predicted to project into the periplasm. Phylogenetic analysis revealed that SMc03872 is highly conserved among alphaproteobacteria (indicated by red lines in Fig. S3). The class of *Alphaproteobacteria*, within the phylum *Proteobacteria*, encompasses *S. meliloti* and other bacterial species capable of entering persistent pathogenic or beneficial host interactions. These alphaproteobacteria also include the chronic, intracellular mammalian pathogen *Brucella abortus* and the plant pathogen *Agrobacterium tumefaciens*.

10.1128/mBio.01060-17.3 FIG S3  Phylogenetic overview of the distribution of SMc03872 homologues across microbial species. Microbesonline.org gene tree tool with a cluster cutoff of ≥70% identification (id) limiting the number of clusters to ≤100. The resulting protein sequences were extracted and imported into bosque 2.0.2 phylogenetic analysis software for alignment and generation of a preliminary phylogenetic tree using the FastTree option. The final tree was visualized by FigTree (v1.4.3). Red lines indicate the presence of conserved LysM motifs. Download FIG S3, PDF file, 0.3 MB. Copyright © 2017 Arnold et al. 2017 Arnold et al. This content is distributed under the terms of the Creative Commons Attribution 4.0 International license.

To gain insights into the importance of *smc03872* during antimicrobial stress and the legume symbiosis, we generated an in-frame chromosomal deletion of the gene, leaving only three N- and C-terminal codons in the chromosome (Δ*smc03872*). We found that the deletion mutant was about 10 times more sensitive to killing by the NCR247 peptide compared to the wild-type strain but could be complemented by a plasmid carrying *smc03872* (Fig. 5D). We introduced two site-directed mutations that affect the conserved lipoprotein residues (IAGCQS) of the plasmid-borne copy of SMc03872 (Fig. 5B). Residues C_29_ and Q_30_ were mutated to serine and aspartate, respectively. The cysteine at this conserved position is important for bacterial lipoproteins to attach key acyl residues to anchor a lipoprotein in bacterial membranes (65). The glutamine (residue 2) is the location-determining amino acid residue. An aspartate in this position results in the retention of a lipoprotein in the inner membrane (63). We found that the C_29_ residue is important for the protective function of SMc03872 against the antimicrobial action of NCR247 (Fig. 5D). In contrast, mutating the Q_30_ residue to aspartate did not result in significant changes in the ability of SMc03872 to protect against the antimicrobial activity of NCR247 (Fig. 5D). These findings suggest that SMc03872 is a lipoprotein whose localization to the outer membrane is not essential for the protein to exert its protective function as long as it is anchored to either the inner or outer membrane.

To investigate the importance of the catalytic activity of the conserved peptidase domain in SMc03872 during NCR247 exposure, we introduced two site-directed mutations into the conserved peptidase motif (H_147_E_148_XXH_151_). The conserved residues H_147_ and E_148_ were changed to arginine and glutamine, respectively (66). Both site-directed mutations, H147R and E148Q, eliminated the ability of SMc03872 to protect the *S. meliloti* Δ*smc03872* mutant from the antimicrobial activity of NCR247 (Fig. 5E). This suggests that the peptidase activity of SMc03872 is crucial for the protein’s protective function against the cationic NCR247 peptide. Furthermore, to establish the importance of the C-terminal LysM domain for the protective functions of SMc03872, we constructed a mutant version of gene *smc03872* encoding a truncated protein lacking the C-terminal 51 amino acids (ΔLysM) (Fig. 5C). The gene, encoding the truncated peptidase was not able to rescue the sensitivity of the Δ*smc03872* mutant to killing by NCR247 (Fig. 5E). To ensure that these results are not due to loss of stability of SMc03872, we also created C-terminally His-tagged versions of the full-length and ΔLysM SMc03872 proteins and expressed them in an *S. meliloti* 1021 Δ*smc03872* strain background. We found that both proteins were expressed (Fig. 5E, inset), and therefore, it was assumed that the loss of protective function of SMc03872 is due to the lack of the predicted LysM domain. Therefore, a functional peptidase domain and the predicted LysM domain are essential for SMc03872 to protect *S. meliloti* against the antimicrobial action of NCR247.

### The putative periplasmic peptidase SMc03872 is important for competitiveness during symbiosis.

Although the *smc03872* gene is not essential for the symbiosis because a deletion mutant is able to support legume growth on nitrogen-free media with no obvious difference in plant size or leaf color (Fig. S4A), we did find that SMc03872 is important for *S. meliloti* competitiveness *in planta*. We inoculated 2-day-old alfalfa (*M. sativa*) seedlings with an approximately 50/50 mixture of wild-type and Δ*smc03872* mutant bacteria (Fig. 5F) and allowed the plants to grow for 3 weeks for nodule development. Then, nodules were randomly selected, and all bacteria that had not lost their ability to divide were recovered by selective plating, utilizing a Δ*smc03872* mutant carrying the pRF771 (Tet^r^) plasmid. We found that Δ*smc03872* mutant bacteria were approximately 100 times less competitive (Fig. 5G). To ensure that plasmid pRF771 that was used to distinguish the mutant strain from wild-type bacteria was actually stable during our plant competition assays, we performed a control experiment with an equal mixture of a wild-type without and another wild-type carrying plasmid pRF771. We found that about 30% of the recovered bacteria carried plasmid pRF771 (Fig. S4B). This observation suggested that the reduction of Δ*smc03872* mutant cells is not due to plasmid loss. When the *smc03872* gene was reintroduced into the deletion mutant by expression from plasmid pRF771, the number of recovered deletion mutant bacteria was more than 10 times larger than the Δ*smc03872* mutant strain when in competition with a wild-type strain (Fig. 5G). Taken together, these observations indicate that *smc03872* is important for *S. meliloti* competitiveness during the symbiosis.

10.1128/mBio.01060-17.4 FIG S4  An *S. meliloti* Δ*smc03872* mutant is proficient in supporting legume growth, and plasmid pRF771 is stable during symbiosis with only minimal plasmid loss. (A) Bacterial composition of alfalfa plant inoculum of the indicated strains. Values are means ± standard deviations (error bars). (B) Bacterial cells recovered from 21-day-old nodules represented by the fraction of wild-type bacterial cells recovered with and without plasmid pRF771. Values means ± standard errors (error bars) for five nodules. Experimental results shown are representative for trends observed in at least two independent experiments. Download FIG S4, PDF file, 1.3 MB. Copyright © 2017 Arnold et al. 2017 Arnold et al. This content is distributed under the terms of the Creative Commons Attribution 4.0 International license.

## DISCUSSION

**FIG 6:**
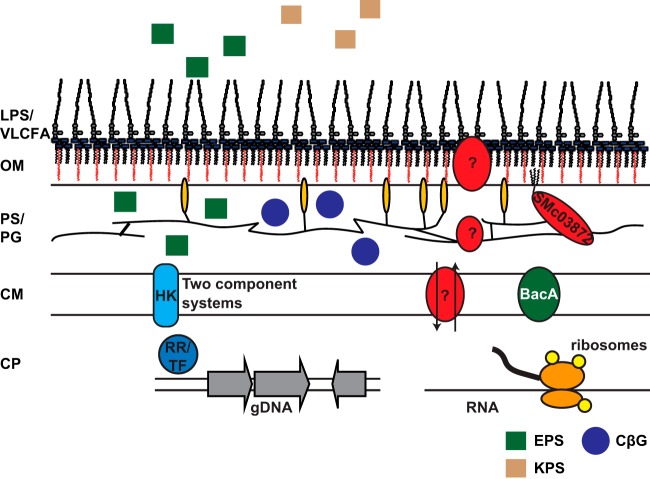
Schematic depiction of the Gram-negative cell envelope highlighting bacterial factors that may affect NCR247 competitiveness. Abbreviations: LPS, lipopolysaccharide; VLCFA, very-long-chain fatty acids; OM, outer membrane; PS, periplasm; PG, peptidoglycan; CM, cytoplasmic membrane; CP, cytoplasm; HK, histidine kinase; RR, response regulator; TF, transcription factor; gDNA, genomic DNA; EPS, extracellular polysaccharides; CβG, cyclic β-glucans; KPS, capsular polysaccharide.

NCR peptides are essential signaling molecules for *Medicago* plant species to trigger the physiological changes in *S. meliloti* required for successful nitrogen fixation (3, 9, 67). A subset of these cysteine-rich peptides are antimicrobial at higher doses, so rhizobia likely possess mechanisms to protect themselves from the antimicrobial action of these NCR peptides (3, 10, 11). In principle, *S. meliloti* cells could protect themselves by modulating the effective dose they experience, for example by altering the characteristics of their cell surface or, alternatively, by inducing mechanisms that counteract specific antimicrobial consequences of NCR exposure. When we began this study, the only *S. meliloti* gene known to influence NCR247 antimicrobial activity was *bacA* (10). Our study has revealed that *S. meliloti* uses a wide variety of mechanisms ranging from modifications of the cell envelope to the utilization of transport systems and cytoplasmic factors to achieve a state in which they can respond appropriately to the symbiotic signaling by the NCR peptides (Fig. 6). Identification of the numerous genes involved in NCR247 sensitivity will allow investigation of their modes of action and their roles during symbiosis. A second key observation is that, while some of the mechanisms that protect against the cationic NCR247 peptide are general enough to protect against the well-studied cationic peptide PMB, such as LPS and extracellular polysaccharide (EPS), other protective mechanisms are specific to either NCR247 or PMB. This suggests that, in addition to generalized forms of protection, evolution may have selected for some resistance mechanisms that help a particular bacterium to withstand particular antimicrobial peptides it encounters in its particular environment. Our results are consistent with previous data that antimicrobial peptides can cause a variety of physiological effects ranging from a reduction of membrane potential and disrupting bacterial cell envelopes to interference with key cytoplasmic processes (68–71).

Cell envelope polysaccharides play critical roles in modulating the antimicrobial effects on bacteria and are the first structures of Gram-negative bacteria that encounter incoming environmental stresses (Fig. 6). Our results show that LPS core-modifying factors are key for the protection of *S. meliloti* against NCR247 antimicrobial action (Fig. 2A and Fig. S2). They also suggest that the *S. meliloti* extracellular polysaccharide succinoglycan exerts protective functions toward NCR247 (Fig. 2B). An earlier study from our lab found that a number of *exo* genes are upregulated by *S. meliloti* upon encountering a sublethal dose of NCR247 (9), and therefore, it is possible that succinoglycan biosynthesis is upregulated by *S. meliloti* in response to encountering cationic NCR peptides in order to protect itself from the antimicrobial actions of cationic NCR peptides among other functions. Due to the two succinate and pyruvate side groups of the succinoglycan octasaccharide subunit carrying net negative charges (31, 72, 73) and NCR247 being a cationic peptide, electrostatic effects between succinoglycan and NCR247 may result in reduced antimicrobial action of NCR peptides on wild-type *S. meliloti*. Paradoxically, some polysaccharides that can contribute positively to symbiosis, such as KPS and CβG, can sensitize the cells to the antimicrobial action of NCR peptides (Fig. 2C and D). This phenomenon may possibly contribute to evolutionary pressure to acquire other mechanisms that can counter this increased sensitivity of the symbiotic function. Succinoglycan is biosynthetically built on an undecaprenol lipid carrier (31) that is also required for the synthesis of other cell envelope components, such as LPS or PG (74), both of which were observed to affect bacterial NCR247 competitiveness in a similar way (Fig. 2 and 3). We found that cellular stress induced by NCR247 is enhanced when later steps of succinoglycan biosynthesis are disabled, while defects in earlier steps (carried out in the cytoplasm) have no strong effect. It is possible that mutations in the early genes halt succinoglycan biosynthesis and the lipid carrier can be recycled to be used in other biosynthetic pathways, whereas this may not be possible if succinoglycan biosynthesis is halted at later stages of the pathway so that lipid-linked intermediates accumulate (31, 74). Taken together, these observations indicate that polysaccharides are key mechanisms of *S. meliloti* to modulate the antimicrobial effects of NCR247.

Gram-negative bacteria like *S. meliloti* have a thin layer of PG between their cytoplasmic and outer membranes, and our findings suggest a protective function of PG during NCR247 exposure (Fig. 3E). During the legume symbiosis, the *S. meliloti* cell envelope requires substantial remodeling as the cells increase in size and change their physiology to prepare for optimal nitrogen fixation, and therefore, PG most likely plays a key role during symbiosis (1, 75). However, *S. meliloti* PG structure and function are still understudied. Here, we show that multiple putative PG-associated factors play a protective role during NCR247 exposure and may also be critical players for successful symbiosis.

We identified the *smc03872* gene encoding a protein with a very intriguing phylogeny that is specific to alphaproteobacteria and provides strong protection against NCR247 and PMB (Fig. 5A and Fig. S3). SMc03872 is a putative outer membrane periplasmic lipoprotein peptidase with the ability to bind periplasmic PG. It is possible that SMc03872 modifies peptide residues of PG to raise basal resistance to cationic NCR peptides and increase symbiotic competitiveness. Alternatively, SMc03872 might exert its peptidase activity degrading cationic peptides such as NCR247 and PMB. A distantly related peptidase in *E. coli* is BepA (39% similarity to SMc03872) (66), which is located in the periplasm. Just like SMc03872, BepA has a conserved M48 peptidase domain, and it plays a role in outer membrane quality control by interacting with the outer membrane protein BamA, but it lacks a LysM domain (76). In this study, we found that two proteins that show homology to the translocation and assembly module (Tam) components A and B, respectively (55) support competitiveness during NCR247 and PMB exposure. Tam systems are structurally related to the essential β-barrel assembly machine (Bam) system that is known to insert β-barrel proteins into bacterial membranes (55, 77). Therefore, mutations in either of these genes may change membrane properties and result in alterations of the bacterial surface which are likely to affect sensitivity to the antimicrobial action of NCR247. Just as in *E. coli* with BepA and BamA, there might be a connection between SMc03872 and the Tam system in *S. meliloti* that still has to be established. For future experiments, it will be worthwhile to test the peptidase mutant with other known NCR peptides (e.g., cationic NCR035) either from *Medicago* plant species or other legumes, such as *Aeschynomene*. Also, it will be interesting to know whether deletion of similar peptidase genes in other rhizobia also mediates sensitivity to killing by NCR247 or other NCR peptides. One of the outcomes of these experiments will help to answer whether the protein encoded by gene *smc03872* is a broad-spectrum peptidase.

The zoologically important, highly infectious alphaproteobacterial pathogen *B. abortus* also has a conserved homologue to this peptidase (approximately 76% similarity), as may other important bacterial pathogens, which makes SMc03872 a potential drug target whose inactivation would result in increased sensitivity to host AMPs. This may also be true for additionally uncharacterized bacterial factors that have been uncovered by our study.

The BacA protein has been proposed to alter transport processes across the inner membrane; however, its physiologically important substrates are currently not known (47, 49). A *bacA* mutant lacks about 50% of its outer membrane VLCFA modifications, and in this study we found that an *acpXL* mutant which lacks 100% of its VLCFA (45, 49) shows decreased competitiveness during NCR247 treatment (Fig. 3A and Fig. S2A). This suggests that VLCFAs provide protection against the antimicrobial action of NCR247. Therefore, it is likely that the reduced amount of VLCFA of a *bacA* mutant does contribute toward its increased sensitivity toward NCR247. However, it has also been proposed that BacA mediates NCR peptide uptake (48) to decrease the effective dose of NCR peptides that potentially harms the cellular membranes. However, the molecular mechanisms that underlie the phenotypes of *S. meliloti bacA* mutants remain elusive. In recent years, BacA has become a paradigm for genes that are important for both the legume symbiosis and also chronic intracellular *B. abortus* (78) and *Mycobacterium tuberculosis* infections (79, 80).

In addition to BacA, we found other membrane transporters that affect the sensitivity to the antimicrobial action of the NCR247 peptide. These systems are probably either involved in transport of cellular substrates into or out of the cytoplasm or might possibly mediate import of NCR247 into the cytoplasm (putative YejABEF ABC transporter) to avoid NCR-mediated damage to bacterial membranes and loss of membrane potential (11, 69). Recently, a homologous ABC transport system in *B. abortus* (53) and *Salmonella* (52) was shown to be important for bacterial virulence and also provides resistance to stresses, such as human defensin peptides, acid pH, or PMB treatment. PMB protection of *S. meliloti* by the YejABEF system was not observed in this study (Fig. 3B). In contrast to BacA and the YejABEF system, the putative *S. meliloti* MLA system that mediates sensitivity toward NCR247 may, just as in *E. coli*, play a key role in maintaining the lipid asymmetry of the outer membrane (54). This finding is in line with our observations that cell envelope structures are key factors that affect NCR247 sensitivity, and further studies into the underlying molecular mechanisms are required to elucidate how the various membrane transporters either protect or sensitize *S. meliloti* toward the antimicrobial action of NCR247.

Our results lead us to speculate that mutations that would tend to retain NCR247 in the periplasm lead to enhanced sensitivity to killing and mutations that would either keep NCR247 outside the cell, or alternatively import it into the cytoplasm, lead to resistance (Fig. 6). For example, negatively charged succinoglycan (extracellular) protects from stress, while negatively charged cyclic β-glucans (periplasmic) will potentiate the stress. Moreover, uptake transporters appear to protect from stress, possibly by removing peptides from the periplasm and delivering it to the cytoplasm where they can be inactivated.

While it is obvious that cell envelope-associated components alter the antimicrobial action of a cationic NCR peptide, the roles of transcriptional regulators and ribosome-associated proteins remain a mystery, and further studies may reveal what biological pathways they affect and how these findings then fit into the complex effects that NCR peptides have on *S. meliloti* and related bacterial species. These studies potentially reveal that newly discovered regulons include aforementioned genes or even genes that cannot be attributed to any pathway at this time (Tables S3 and S4).

10.1128/mBio.01060-17.9 TABLE S4  Bacterial strains and plasmids used in this study. Download TABLE S4, DOCX file, 0.02 MB. Copyright © 2017 Arnold et al. 2017 Arnold et al. This content is distributed under the terms of the Creative Commons Attribution 4.0 International license.

The *E. coli* acetyltransferases RimI, RimJ, and RimL acetylate the N termini of ribosomal proteins S18, S5, and L12, respectively (59, 60). RimL has been shown to acetylate McC lysine residues, resulting in the inactivation of this otherwise toxic peptide-nucleotide antibiotic (81). Two putative *S. meliloti* acetyltransferases were identified that show homology to *E. coli* Rim acetyltransferases (Fig. 4B). Although there are no canonical lysine acetylation sites present in the NCR247 peptide (10, 12), it is possible that NCR247 is modified by one of these acetyltransferases by N-terminal acetylation. Our results indicate that these *S. meliloti* acetyltransferases play distinct roles during NCR247 exposure, as RimI (SMc02449) appears to play a protective role while RimJ/L (SMc00075) appears to play a sensitizing role, suggesting the presence of finely tuned regulation mechanisms employed by *S. meliloti* to regulate sensitivity toward NCR247. Together with the discovery that PNPase with its rRNA processing activity affects NCR247 sensitivity, this suggests that ribosomes, or at least ribosome modifications, play a critical role in modulating the antimicrobial action of NCR247 activity (62). In fact, a previous study, performing pulldown experiments with a labeled NCR247 peptide found multiple ribosomal proteins to be interacting with NCR247 (67). The same study also found that NCR247 is able to inhibit bacterial translation (67).

Our findings suggest that multiple bacterial mechanisms modulate the antimicrobial action of cationic, host defensin-like peptides in the intracellular symbiont *S. meliloti*. The same may be true for mammalian defensins and their interactions with members of other bacterial species. Future work is necessary to investigate the functions of previously uncharacterized genes that affect bacterial sensitivity to antimicrobial host cysteine-rich peptides. This Tn-seq data could be complemented by future transcriptome sequencing (RNA-seq) analysis under similar conditions or RNA-seq analysis of the transcriptional regulator mutants identified in this study to identify and characterize their putative regulons.

Some of these discoveries will have the potential to become drug targets to support innate antimicrobial peptide activity in fighting bacterial pathogens. Our experimental observations open up the field of host-microbe interactions to a new set of genes that may play crucial roles in symbiosis and related persistent host-microbe interactions.

## MATERIALS AND METHODS

### Bacterial culture and strains.

The bacterial strains and plasmids used in this study are summarized in Table S4 in the supplemental material. *S. meliloti* strains were grown in lysogeny broth (LB) (5 mg ml^−1^ NaCl) (82) supplemented with 2.5 mM MgSO_4_ and 2.5 mM CaCl_2_ (LBMC) and 200 µg ml^−1^ streptomycin (Sm) at 30°C for 48 h. If required, *S. meliloti* cultures were supplemented with additional antibiotics. *E. coli* strains were grown in LB at 37°C for 17 h and supplemented with the indicated antibiotics, if required. The following antibiotics were used at the indicated concentrations: tetracycline (Tet), 10 µg ml^−1^; gentamicin (Gent), 50 µg ml^−1^; neomycin (Neo), 100 µg ml^−1^.

### Transposon insertion library preparation.

A HIMAR1 transposon (19) library in *S. meliloti* 1021 was created using triparental mating. Wild-type *S. meliloti* (recipient), *E. coli* MT616 (helper), and *E. coli* Sm10λpir (83) carrying pSAM_DGm (84) (donor) were grown to late exponential phase and then diluted to an optical density at 600 nm (OD_600_) of 2, 1, and 1, respectively. Then, the cultures of the defined optical densities were mixed in equal proportions, transferred onto LBMC, and incubated at 30°C for 6 h. The resulting colonies were then collected in saline (0.85% NaCl) and spread equally onto 50 large petri dishes (150 by 15 mm; VWR) containing LBMC Sm Gent agar (LBMC agar supplemented with Sm and Gent) and incubated at 30°C for 72 h to obtain transposon mutants represented by single colonies. All colonies from all 50 large petri dishes were pooled into saline, concentrated using a Sorvall centrifuge (Thermo Scientific Lynx 4000), and stored in aliquots at −80°C after the addition of glycerol to a final concentration of 20%.

### NCR247 peptide preparation.

The legume nodule-specific cysteine-rich peptide NCR247 was synthesized as described previously in the reduced form and subsequently oxidized overnight under aerobic conditions in the presence of 20% dimethyl sulfoxide (DMSO) (12). For all experiments in this study, NCR247 was used as a mix of all three regio-isomers in the thermodynamically stable distribution (NCR247-AR).

### Screen for mutant competitiveness during antibiotic stress.

The transposon mutant library was inoculated into 500 ml of LBMC Sm Gent at an OD_600_ of 0.001 and incubated with continuous shaking until the culture reached an OD_600_ between 0.1 and 0.3. This high volume of an initial inoculation was necessary to ensure that as many as transposon insertion mutants as possible were represented in the culture. At this point, 50 ml was spun down and resuspended in 1 ml of prewarmed 3-(*N*-morpholino)propanesulfonic acid (MOPS)-buffered minimal medium (50 mM MOPS, 1 mM MgSO_4_, 0.25 mM CaCl_2_, 19 mM glutamic acid, 4 mM biotin [pH 7.0]) supplemented with 1% Casamino Acids (MOPS-GS Cas) and washed two times in the same buffer. Then, the cells were resuspended to an OD_600_ of 0.1 in 10 ml of fresh MOPS-GS Cas (cell density of approximately 16,000 cells per mutant), and NCR247-AR was added to a final concentration of 6 µM or polymyxin B (PMB) was added to a final concentration of 0.8 µg ml^−1^. All treated and untreated cultures were then incubated at 30°C for 10 h with continuous shaking (200 rpm), spun down, and resuspended for retreatment with the same amount of antibiotic peptide and incubated for an additional 10 h (PMB) or 12 h (NCR247-AR). All experimental cultures concluded two to four doublings as shown by a representative growth pattern analysis (Fig. S5A). The concentration of 6 µM was chosen for NCR247-AR, because under our assay conditions, it resulted in initial killing of wild-type *S. meliloti* 1021 cells followed by resumption of bacterial growth (Fig. S5A). We were anticipating that this effect would increase the effects that we would observe. The concentration of 0.8 µg ml^−1^ was chosen for PMB, as it allowed slow growth of *S. meliloti* 1021 cells under our assay condition (Fig. S5A). For PMB, we were not able to find a concentration under which cell killing occurred followed by recovery like we observed for NCR247-AR. The number of doublings was determined by the cell viability numbers obtained before peptide treatments and after outgrowth of the transposon mutant populations (Fig. S5A).

10.1128/mBio.01060-17.5 FIG S5  Representative *S. meliloti* growth dynamics and reproducibility during the Tn-seq experiments. (A) Representative *S. meliloti* killing and growth dynamics during the Tn-seq experiments. The plots show representative data for the growth dynamic of the *S. meliloti* transposon mutant library during each experimental condition. (B) Reproducibility of normalized read count per gene between the two biological replicates. Download FIG S5, PDF file, 4.6 MB. Copyright © 2017 Arnold et al. 2017 Arnold et al. This content is distributed under the terms of the Creative Commons Attribution 4.0 International license.

All experimental cultures, including an untreated culture derived from the mutant library pool, were collected and prepared for Illumina sequencing. The nature of the transposon with its flanking MmeI restriction sites allowed us to uniquely map the majority of transposon insertions by sequencing the genomic DNA (gDNA) (16 bp) adjacent to each transposon (19). A similar approach was used in a previous study in related rhizobia (21). Two biological replicates were carried out for the described experiment.

### Preparation of sample DNA for sequencing.

Genomic DNA from treated and untreated *S. meliloti* transposon mutant libraries was isolated using a GenElute bacterial genome kit (Sigma). Then, 10 µg of gDNA was digested with MmeI (New England Biolabs) according to the manufacturer’s instructions for 1 h followed by 1-h treatment with 5 µl (10,000 U/ml) of calf intestinal alkaline phosphatase (CIP) (New England Biolabs). After DNA purification (Qiagen), the digested gDNA was ligated with double-stranded adapter DNA.

Double-stranded adapter DNA was prepared beforehand by mixing single-stranded adapters 1 and 2 (a final concentration of 0.5 µM for each adapter) in Qiagen elution buffer supplemented with 6 mM NaCl and 300 µM MgCl_2_. This reaction mixture was incubated at 95°C for 5 min and then allowed to slowly cool down (0.1°C/s). Double-stranded adapter molecules were ligated to MmeI-digested gDNA as follows using a LigaFast rapid ligation kit (Promega). A typical reaction mixture contained 12 µl of gDNA, 15 µl of T4 DNA ligation buffer (2×), 2 µl of T4 Quick ligase, and 1.2 µl of 2.4 µM double-stranded adapter.

The resulting MmeI-digested gDNA with ligated adapter (2 µl) was then enriched with PCR using NEB Phusion DNA polymerase according to the manufacturer’s instructions. A universal transposon primer and the corresponding barcoded primers were used for each reaction. These primers contained necessary anchor sequences for Illumina flow cell hybridization. Sequencing was performed using a HiSeq instrument (Illumina) using the barcode and universal primers as shown in Table S5. A comprehensive list of the primers used for sequencing is listed in Table S5.

10.1128/mBio.01060-17.10 TABLE S5  Primers used in this study. Download TABLE S5, DOCX file, 0.01 MB. Copyright © 2017 Arnold et al. 2017 Arnold et al. This content is distributed under the terms of the Creative Commons Attribution 4.0 International license.

### Sequencing data analysis.

Data analysis was performed using a custom package called TnSeq-Pipeline. TnSeq-Pipeline is a lightweight python script that facilitates the analysis of transposon sequence data, from fastq files to tallied transposon counts across a user-supplied reference genome. It uses Bowtie 2 (85) for read mapping and currently supports analysis of single end reads and an arbitrary number of experimental conditions. It supports fuzzy transposon matching, reverse complementing input reads, read normalization, and the removal of transposons located in the edges of genes to ensure that only transposon events which lead to functional knockout are included in the analysis. Users configure these and other settings in a configuration file prior to running the program. A typical TnSeq-Pipeline run consists of three steps: prealignment, alignment, and postalignment processing. In the prealignment stage, fasta/fastq files are processed, transposon end sequences are trimmed, and sequences not containing the transposon sequence are removed. Reads containing the transposon pass onto the alignment stage. Alignment is accomplished using Bowtie 2 with standard settings. As such, this pipeline requires the user to install Bowtie 2 and create a special indexed reference file for their genome of interest. The third stage involves processing the SAM file output of Bowtie 2. With user-supplied ptt files of the reference genome, this process yields both transposon insertion event summaries for individual sites and for whole genes. TnSeq-Pipeline also provides output compatible with IGV (Integrative Genomics Viewer) (86, 87), allowing researchers to visualize transposon insertion density across the genome across different conditions. TnSeq-Pipeline is distributed under the MIT license. Information on downloading, installing, and running TnSeq-Pipeline can be found on GitHub (https://github.com/KBoehme/TnSeq-Pipeline).

To identify Illumina reads that contained the transposon sequence, we identified reads with the sequence CGATCTAGACCGGGGACTTATCATCCAACCTG allowing for three mismatches for finding the transposon sequence and then aligned the 17 nucleotides adjacent to it that correspond to genomic DNA. No mismatches were allowed for alignment of genomic sequences. Sequencing data were normalized using the number of reads that align between annotated genes (intergenic) by determining a normalization factor for each sequenced sample based on the sample with the lowest number of intergenic reads (Table 1). While processing raw data using TnSeq-Pipeline, to increase the stringency of our analysis, we ignored the effects of transposon insertions within the first and last 10% of each given open reading frame (ORF) sequence because insertion mutations in these regions might not have a detrimental effect on gene function and could possibly result in misleading results (88). Genes that contained transposon insertions with a total normalized read count representation of less than 100 in either biological replicate in the starting culture were not considered in this study. Additionally, genes in which transposon insertions caused a loss of competitiveness of more than twofold in the untreated culture were not considered for further analysis.

In total, each sequenced culture condition was represented by between 11 million and 14 million read counts in annotated ORFs. To ensure comparability between samples and replicates, all sequenced cultures across both biological replicates were normalized by TnSeq Pipeline, resulting in each condition being represented by close to 11 million total reads. For analysis purposes, the number of reads from every transposon insertion within a given gene was combined and then expressed as a total number of reads per gene. The readouts appeared highly reproducible between both biological replicates (Fig. S5B).

### Generation of the *smc03872* deletion mutant and complementation constructs.

*S. meliloti* deletion mutant Δ*smc03872* was prepared as previously described (89) with modification. The upstream and downstream flanking regions (approximately 500 bp) of the gene were amplified using primers *smc03872*_del_M_A_R and *smc03872*_del_A_F (del stands for deletion, R stands for reverse, and F stands for forward) and primers *smc03872*_del_M_B_F and *smc03872*_del_B_R, respectively. The resulting DNA fragments were joined by overlap extension PCR using the primers *smc03872*_del_A_F and *smc03872*_del_B_R, followed by BamHI/XbaI digestion and ligation into pK18MobSacB which was subsequently transformed into *S. meliloti* 1021 using overnight triparental mating with helper strain *E. coli* MT616 followed by plating on selective LBMC Sm Neo medium, and then four colonies were restreaked on LBMC Sm sucrose (5%) medium. *S. meliloti* gene deletion mutants were screened for the loss of the pK18MobSacB plasmid by their inability to grow on LBMC supplemented with Sm or Neo.

### Generation of vector constructs for complementation and gene expression experiments with *smc03872*.

Complementation constructs for the mutants created were generated as previously described (46, 80). In brief, genes for complementation were cloned into the PstI and XbaI sites of pRF771 using primer *smc03872_Nsi_F* as a forward primer. Depending on the gene version, primers *smc03872_Xba_R* (full-length gene), *smc03872_Lys-_XbaI_R* (truncated gene), *smc03872_His_XbaI_R* (full-length gene with His tag), *smc03872_ΔLys_His_XbaI_R* (truncated gene with His tag).

For the generation of site-directed mutations in Smc03872, gene *smc03872* was cloned into the EcoRI and HindIII restriction sites of expression vector pET22b(+) (Novagen) after amplification from *S. meliloti* gDNA using primers *smc3872*_EcoRI_ F and *smc3872*_HindIII_R to create pET*smc03872*.

### Generation of site-directed mutations in *smc03872*.

Site-directed mutations in *smc03872* were generated using an Agilent QuikChange XL kit by following the manufacturer’s instructions. To introduce the amino acid changes Q29D, C30S, H147R, and E148Q into SMc03872, the corresponding primer pairs as shown in Table S5 were used on pET*smc03872* as a template.

### Viability experiments for mutant validations.

The defined *S. meliloti* 1021 strains were grown in LBMC to early exponential phase (OD_600_ between 0.1 and 0.3), washed three times in 0.85% saline, and then resuspended to a final OD of 0.1 for treatment with 6 µM NCR247-AR in a final volume of 500 µl in 15-ml Falcon tubes. Reaction mixtures were incubated with shaking for up to 24 h. The number of viable cells for every reaction mixture was then determined by serially diluting and spotting 10-µl aliquots in triplicates on LBMC Strep agar plates.

### Alfalfa interactions and competition experiments.

Three-day-old alfalfa seedlings were inoculated with *S. meliloti* on Jensen’s agar exactly as described previously (90). Single seedlings were planted into the plant medium substrate and inoculated with 1 ml of distilled H_2_O (dH_2_O) washed wild-type/mutant (1:1) *S. meliloti* (final OD_600_ of 0.05). Inoculated plants were kept for the stated length of time at 25°C with a light/dark cycle of 16/8 h, respectively.
