# Supplementary figures and images for: Genome-Wide Sensitivity Analysis of the Microsymbiont Sinorhizobium meliloti to Symbiotically Important, Defensin-Like Host Peptides
Source: mBio. 2017 Aug 1;8(4):e01060-17. doi: 10.1128/mBio.01060-17 (PMC5539429; doi:10.1128/mBio.01060-17)

Figure S1

A

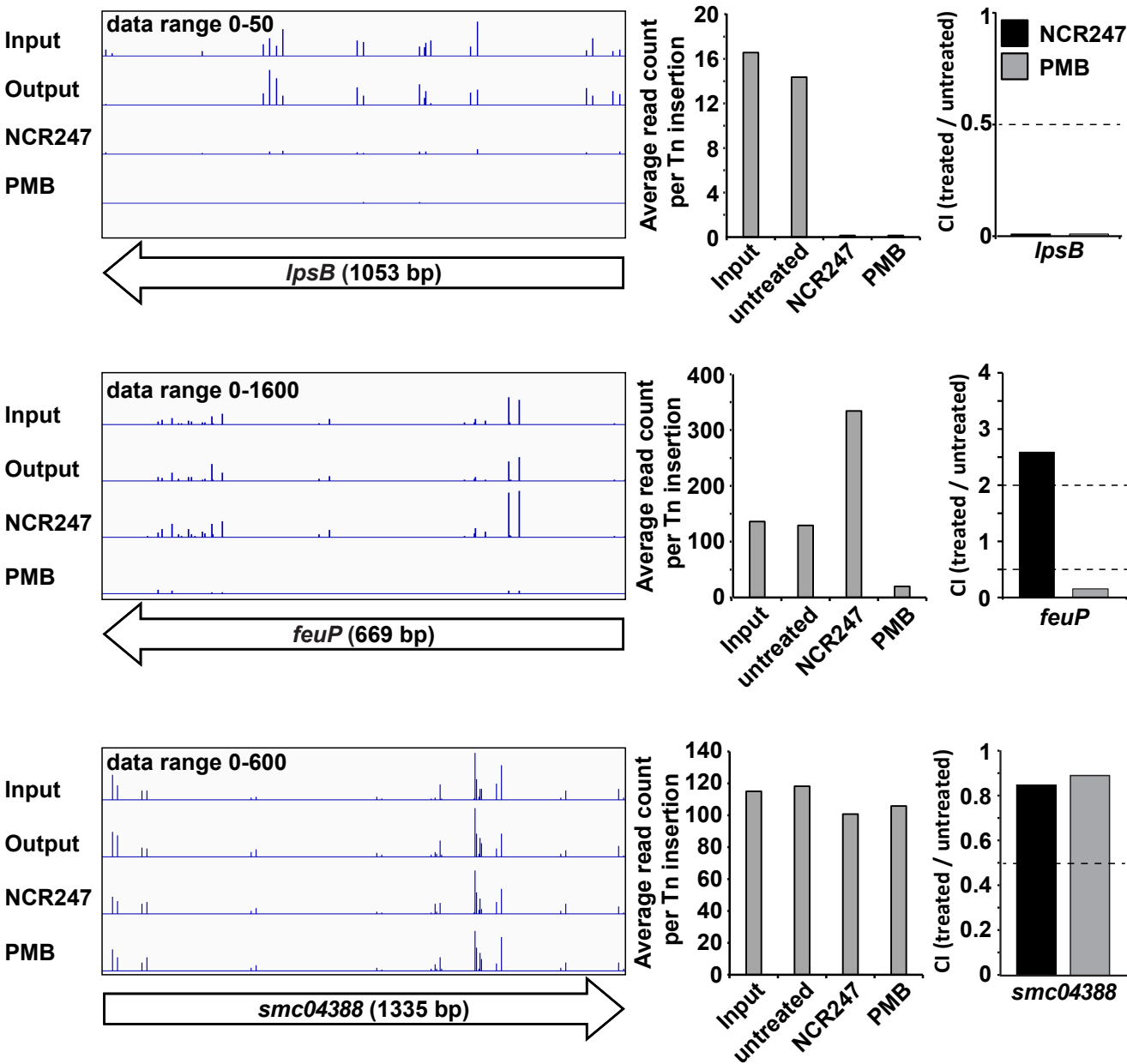

B

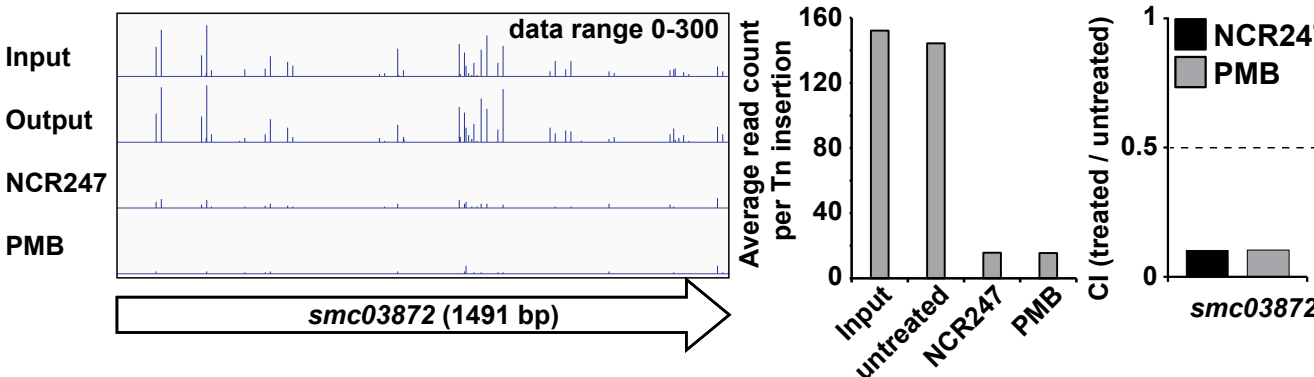

Supplement: FIG S1 [file mbo004173412sf1.pdf]

**Figure S2**

**A** One dose of NCR247AR (10 hour time point)

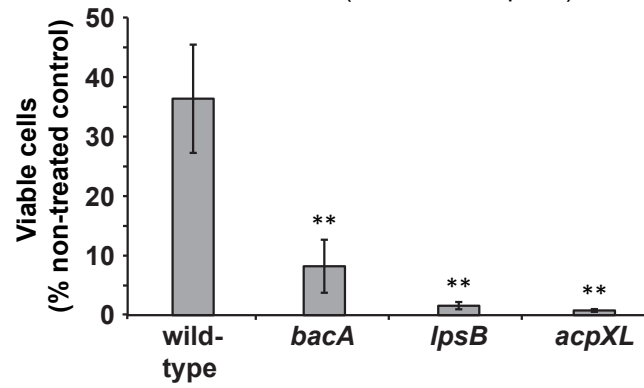

**B** Two doses of NCR247AR (24 hour time point)

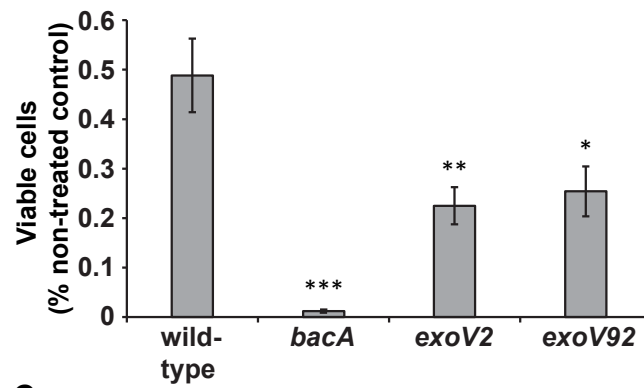

**C**

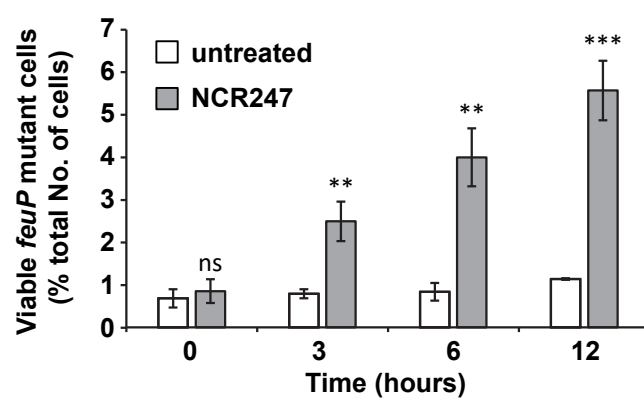

Supplement: FIG S2 [file mbo004173412sf2.pdf]

Figure S3

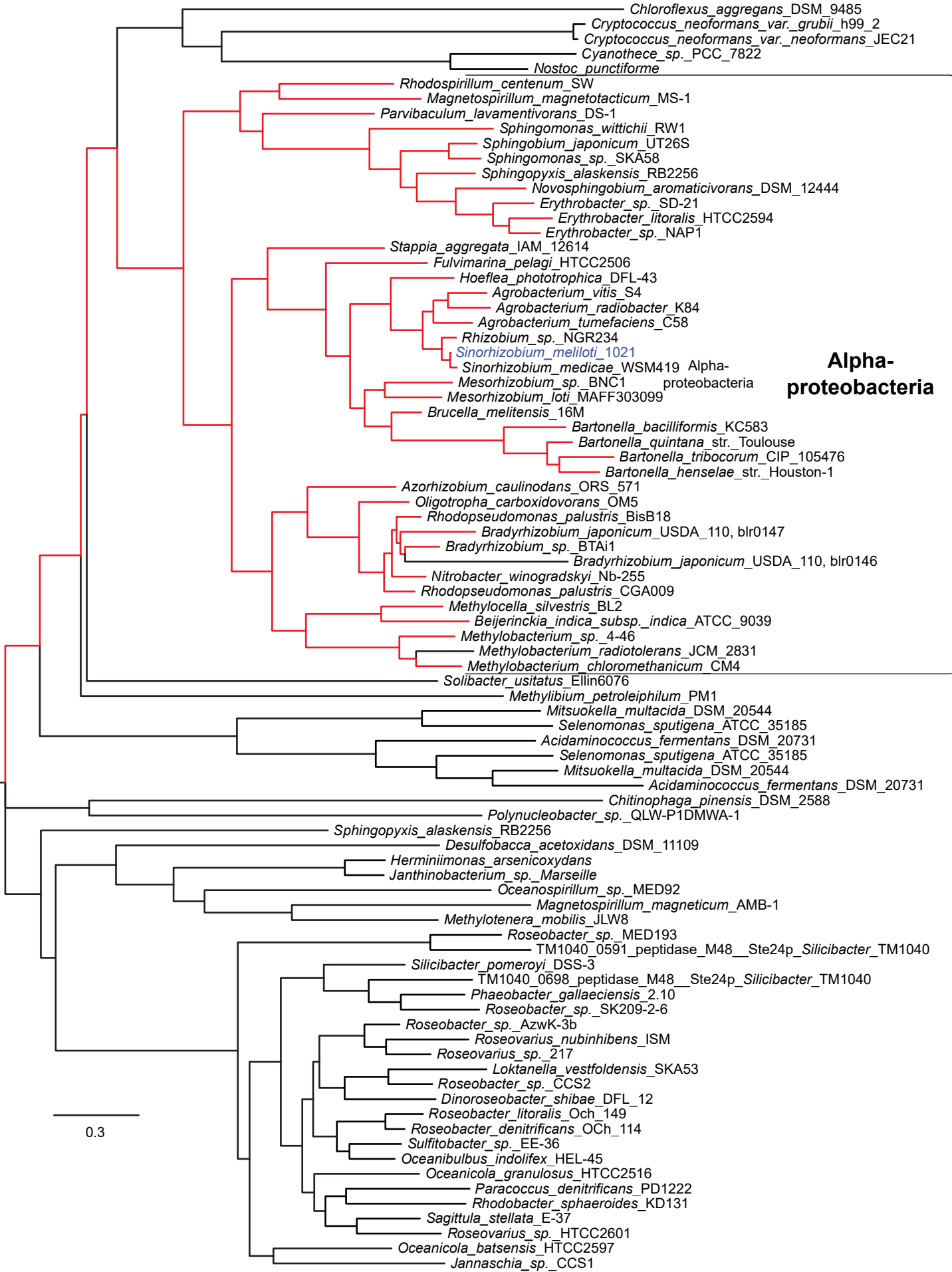

Supplement: FIG S3 [file mbo004173412sf3.pdf]

**Figure S5**

**A**

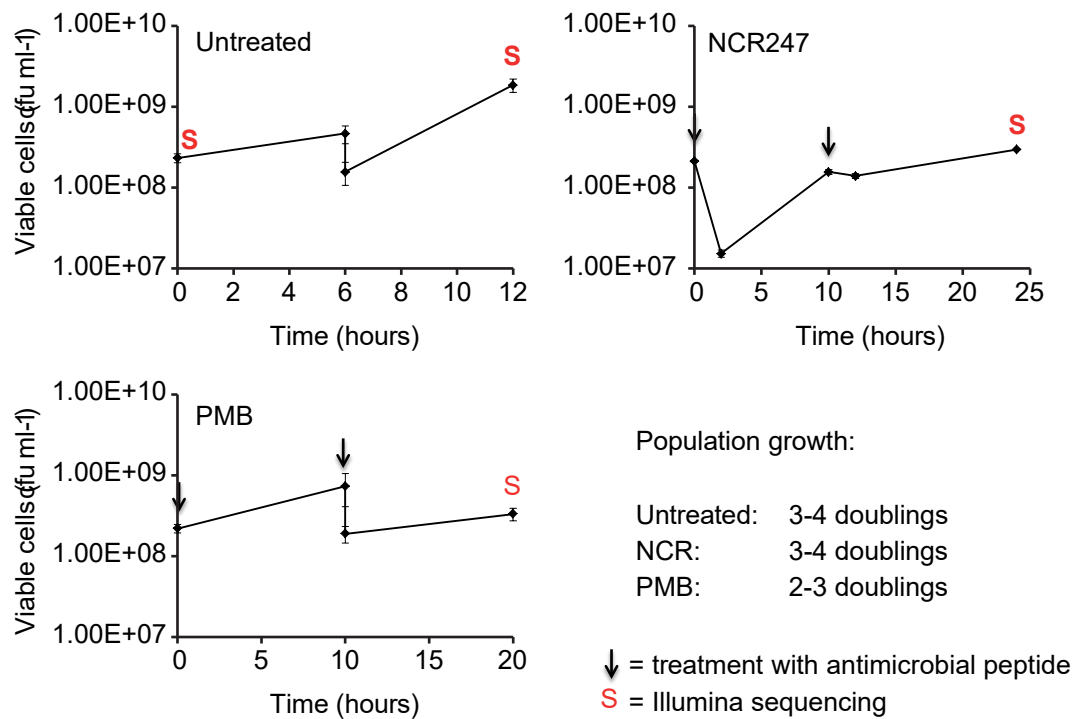

**B**

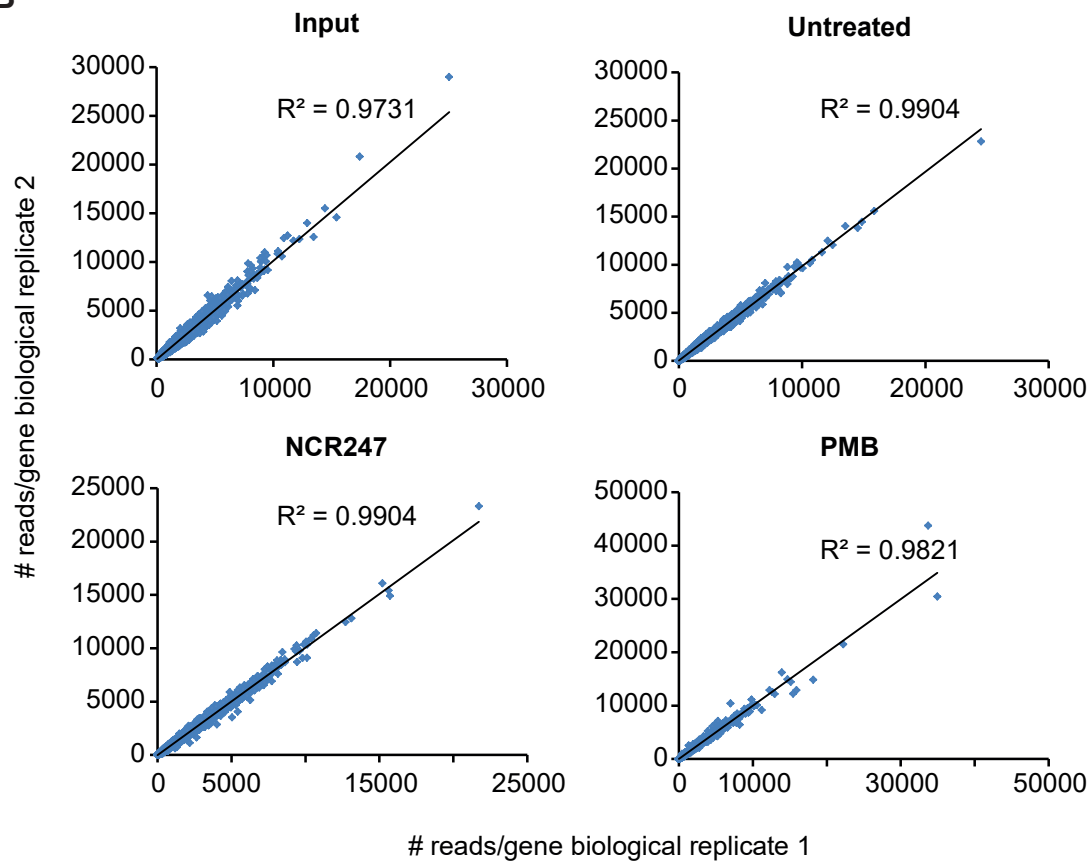

Supplement: FIG S5 [file mbo004173412sf5.pdf]
