# Supplementary material for: Genome-Wide Sensitivity Analysis of the Microsymbiont Sinorhizobium meliloti to Symbiotically Important, Defensin-Like Host Peptides
Source: mBio. 2017 Aug 1;8(4):e01060-17. doi: 10.1128/mBio.01060-17 (PMC5539429; doi:10.1128/mBio.01060-17)
Supplement: FIG S4 [file mbo004173412sf4.pdf]

Figure S4

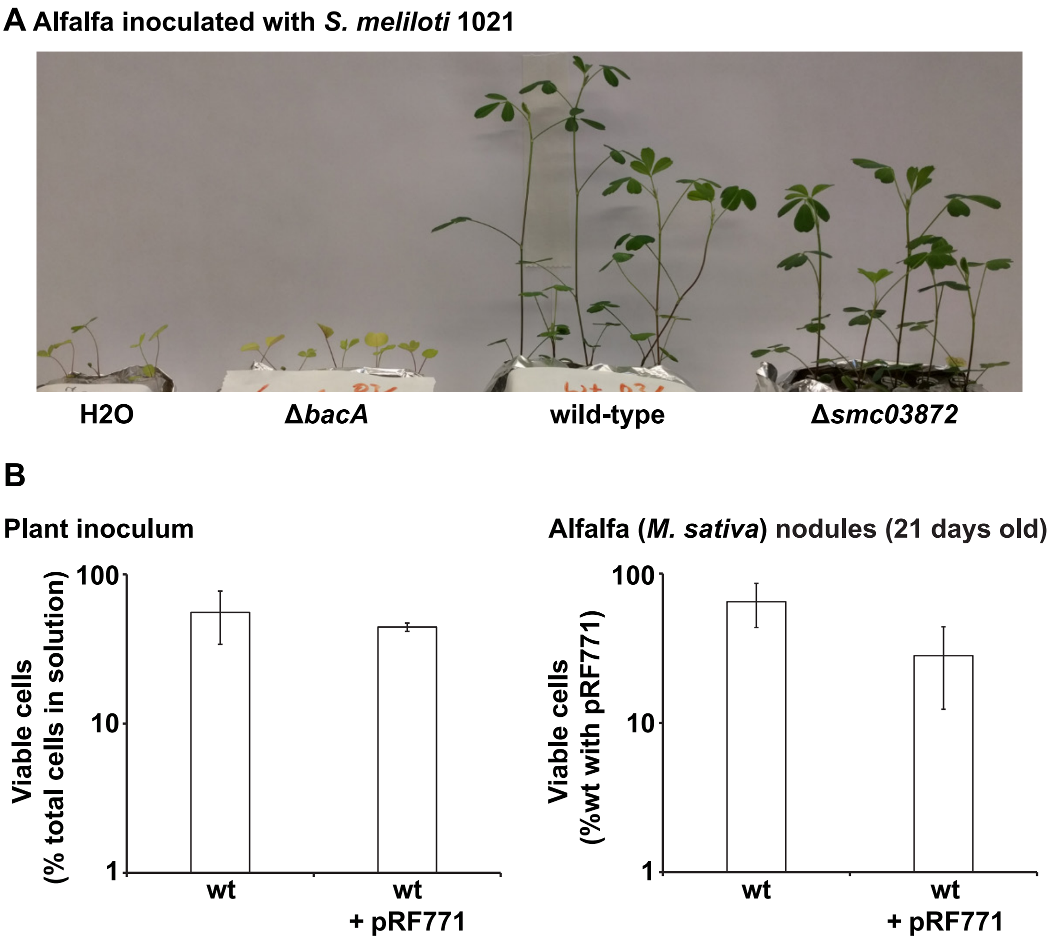

A Alfalfa inoculated with S. meliloti 1021
H2O
ΔbacA
wild-type
Δsmc03872
B
Plant inoculum
Viable cells
(% total cells in solution)
100
10
1
wt
wt
+ pRF771
Alfalfa (M. sativa) nodules (21 days old)
Viable cells
(%wt with pRF771)
100
10
1
wt
wt
+ pRF771
