# Supplementary material for: Genome-Wide Sensitivity Analysis of the Microsymbiont Sinorhizobium meliloti to Symbiotically Important, Defensin-Like Host Peptides
Source: mBio. 2017 Aug 1;8(4):e01060-17. doi: 10.1128/mBio.01060-17 (PMC5539429; doi:10.1128/mBio.01060-17)
Supplement: TABLE S4 [file mbo004173412st4.docx]

**Table S4 Bacterial strains and plasmids used in this study**

| **Strain or Plasmid** | **Relevant characteristic** | **Source or reference** |
| --- | --- | --- |
| ***Sinorhizobium meliloti*** |  |  |
| *S. meliloti* 1021 | wild-type, SmR derivative of SU47 | ([1](#_ENREF_1)) |
| *S. meliloti* 1021 Δ*smc03872* | *Sm*1021 with clean deletion of the *smc03872* gene | this study |
| *S. meliloti* 1021 *lpsb::TnphoA* | *Sm*1021 with TnphaA insertion in gene *lpsB* | ([2](#_ENREF_2)) |
| *S. meliloti* 1021 *acpXL::pK18mobGII* | pK18mobGII plasmid insertion in the *Sm*1021 *acpXL* gene | ([3](#_ENREF_3)) |
| *S. meliloti* 1021 *exoV2::Tn5* | Tn5 insertion in the *Sm*1021 *exoV* gene | ([4](#_ENREF_4)) |
| *S. meliloti* 1021 *exoV98::Tn5* (Rm8287) | Tn5 insertion in the *Sm*1021 *exoV* gene | ([4](#_ENREF_4)) |
| *S. meliloti* 1021 Δ*feuP* | *Sm*1021 with clean deletion of the *feuP* gene | ([5](#_ENREF_5)) |
| *S. meliloti* 1021 Δ*bacA* | *Sm*1021, Δ*bacA*654::SpR | ([6](#_ENREF_6)) |
|  |  |  |
| ***Escherichia coli*** |  |  |
| *E. coli* Sm10λpir | KmR, *thi-1, thr, leu, tonA, lacY, supE, recA*::RP4-2-Tc::Mu, *pir* | ([7](#_ENREF_7)) |
| DH5α | *supE44* Δ*lacU169* (ɸ80lacZΔM15) *hsdR17 recA1 endA1 gyrA96 thi-1 relA1* | Bethesda Research Laboratories |
| MT616 | MM294A *recA56* (pRK600) Cmp^r^ | ([8](#_ENREF_8)) |
|  |  |  |
| **Plasmids** |  |  |
| pSAM_DGm |  | ([9](#_ENREF_9)) |
| pRF771 | RK2 derivative P*trp* expression vector Tet^r^ | ([10](#_ENREF_10)) |
| pK18MobSacB | Frequently used gene knockout plasmid | ([11](#_ENREF_11)) |
| p*smc03872* | pRF771 carrying the entire *S. meliloti smc03872* gene, TetR | This study |
| p*smc03872*-His | pRF771 carrying the entire *S. meliloti smc03872* gene with a C-terminal 6x His tag, TetR | This study |
| p*smc03872*ΔLysM | pRF771 carrying a truncated *S. meliloti smc03872* gene lacking its conserved C-terminal domain (51 amino acids), TetR | This study |
| p*smc03872*ΔLysM-His | pRF771 carrying a truncated *S. meliloti smc03872* gene lacking its conserved C-terminal domain (51 amino acids) with a C-terminal 6x His tag, TetR | This study |
| p*smc03872_*C29S | pRF771 carrying the entire *S. meliloti smc03872* gene with site-directed mutation C29S, TetR | This study |
| p*smc03872_*Q30D | pRF771 carrying the entire *S. meliloti smc03872* gene with site-directed mutation Q30D, TetR | This study |
| p*smc03872_*H147R | pRF771 carrying the entire *S. meliloti smc03872* gene with site-directed mutation H147R, TetR | This study |
| p*smc03872_*E147Q | pRF771 carrying the entire *S. meliloti smc03872* gene with site-directed mutation E147Q, TetR | This study |

**References**

1. Meade HM, Long SR, Ruvkun GB. 1982. Physical and genetic characterization of symbiotic and auxotrophic mutants of *Rhizobium meliloti* induced by transposon Tn5 mutagenesis. J Bacteriol 149:114-122.

2. Campbell GR, Reuhs BL, Walker GC. 2002. Chronic intracellular infection of alfalfa nodules by *Sinorhizobium meliloti* requires correct lipopolysaccharide core. Proc Natl Acad Sci U S A 99:3938-3943.

3. Ferguson GP, Datta A, Carlson RW, Walker GC. 2005. Importance of unusually modified lipid A in *Sinorhizobium* stress resistance and legume symbiosis. Mol Microbiol 56:68-80.

4. Glucksmann MA, Reuber TL, Walker GC. 1993. Genes needed for the modification, polymerization, export, and processing of succinoglycan by *Rhizobium meliloti*: a model for succinoglycan biosynthesis. J Bacteriol 175:7045-7055.

5. Griffitts JS, Carlyon RE, Erickson JH, Moulton JL, Barnett MJ, Toman CJ, Long SR. 2008. A *Sinorhizobium meliloti* osmosensory two-component system required for cyclic glucan export and symbiosis. Mol Microbiol 69:479-490.

6. Ferguson GP, Roop RM, Walker GC. 2002. Deficiency of a *Sinorhizobium meliloti bacA* mutant in Alfalfa symbiosis correlates with alteration of the cell envelope. J Bacteriol 184:5625-5632.

7. Miller VL, Mekalanos JJ. 1988. A novel suicide vector and its use in construction of insertion mutations: osmoregulation of outer membrane proteins and virulence determinants in *Vibrio cholerae* requires *toxR*. J Bacteriol 170:2575-2583.

8. Finan TM, Hartwieg E, LeMieux K. 1984. General transduction in *Rhizobium meliloti*. J Bacteriol 159:120-124.

9. Goodman AL, McNulty NP, Zhao Y, Leip D, Mitra RD, Lozupone CA, Knight R, Gordon JI. 2009. Identifying genetic determinants needed to establish a human gut symbiont in its habitat. Cell Host Microbe 6:279-289.

10. Wells DH, Long SR. 2002. The *Sinorhizobium meliloti* stringent response affects multiple aspects of symbiosis. Mol Microbiol 43:1115-1127.

11. Schafer A, Tauch A, Jager W, Kalinowski J, Thierbach G, Puhler A. 1994. Small mobilizable multi-purpose cloning vectors derived from the *Escherichia coli* plasmids pK18 and pK19: selection of defined deletions in the chromosome of *Corynebacterium glutamicum*. Gene 145:69-73.
