# Supplementary material for: Genome-Wide Sensitivity Analysis of the Microsymbiont Sinorhizobium meliloti to Symbiotically Important, Defensin-Like Host Peptides
Source: mBio. 2017 Aug 1;8(4):e01060-17. doi: 10.1128/mBio.01060-17 (PMC5539429; doi:10.1128/mBio.01060-17)
Supplement: TABLE S5 [file mbo004173412st5.docx]

**Table S5 Primers used in this study**

| **Primer** | **Sequence** |
| --- | --- |
| *Adapter 1* | ACACTCTTTCCCTACACGACGCTCTTCCGATCTNN |
| *Adapter 2* | 5Phos/AGATCGGAAGAGCGTCGTGTAGGGAAAGAGTGT/3Phos |
| *Universal primer* | AATGATACGGCGACCACCGAGATCTACACTCTTTCCCTACACGACGCTCTTCCGATCT |
| *Barcode 1* | CAAGCAGAAGACGGCATACGAGAT**AGTCAG**GTGACTGGAGTTCAGACGTGTGCTCTTCCGATCTAGACCGGGGACTTATCATCCAACCTGT |
| *Barcode 2* | CAAGCAGAAGACGGCATACGAGAT**GACTGA**GTGACTGGAGTTCAGACGTGTGCTCTTCCGATCTAGACCGGGGACTTATCATCCAACCTGT |
| *Barcode 3* | CAAGCAGAAGACGGCATACGAGAT**TCGATC**GTGACTGGAGTTCAGACGTGTGCTCTTCCGATCTAGACCGGGGACTTATCATCCAACCTGT |
| *Barcode 4* | CAAGCAGAAGACGGCATACGAGAT**CTAGCT**GTGACTGGAGTTCAGACGTGTGCTCTTCCGATCTAGACCGGGGACTTATCATCCAACCTGT |
| *Barcode 5* | CAAGCAGAAGACGGCATACGAGAT**ACAGTC**GTGACTGGAGTTCAGACGTGTGCTCTTCCGATCTAGACCGGGGACTTATCATCCAACCTGT |
| *Barcode 6* | CAAGCAGAAGACGGCATACGAGAT**GTGACT**GTGACTGGAGTTCAGACGTGTGCTCTTCCGATCTAGACCGGGGACTTATCATCCAACCTGT |
| *Barcode 7* | CAAGCAGAAGACGGCATACGAGAT**TGCTAG**GTGACTGGAGTTCAGACGTGTGCTCTTCCGATCTAGACCGGGGACTTATCATCCAACCTGT |
| *Barcode 8* | CAAGCAGAAGACGGCATACGAGAT**CATCGA**GTGACTGGAGTTCAGACGTGTGCTCTTCCGATCTAGACCGGGGACTTATCATCCAACCTGT |
| *Barcode read* | AGATCGGAAGAGCACACGTCTGAACTCCAGTCAC |
| *gDNA read* | ACACTCTTTCCCTACACGACGCTCTTCCGATCT |
| *smc03872_del_M_A* | GCCTCACTCCGAGCCATTCATGAC |
| *smc03872_del_M_B* | GTCATGAATGGCTCGGAGTGAGGC |
| *smc03872_del_A_F* | GGAAAAtctagaATGATCACACATCATCCCGATGACGTG |
| *smc03872_del_B_R* | GGAAAAggatccAGCGATCTGATCGACGGCGA |
| *smc03872_C1_F* | CGATCTGCACGAGGAGGAAGG |
| *smc03872_C2_R* | CCTCTGCCCGACGAGCA |
| *smc03872_Nsi_F* | GCTAGAATGCATGAAACGAGAGTGCCGTCCCCATGAATGGCTGGTTTCCGCGG |
| *smc03872_Xba_R* | GGAAAATCTAGATCACTCCGAAATGATCTTCACCTTGTCG |
| *smc03872_His_XbaI_R* | GGAAAATCTAGATCAatggtgatggtgatggtgCTCCGAAATGATCTTCACCTTGTCG |
| *smc03872_ΔLys_XbaI_R* | GGAAAATCTAGATTATCAGACGCGCAGCGGCTTC |
| *smc03872_ΔLys_His_XbaI_R* | GGAAAATCTAGATTATCAatggtgatggtgatggtgGACGCGCAGCGGCTTC |
| *smc03872_Nsi_F* | GCTAGAATGCATGAAACGAGAGTGCCGTCCCCATGAATGGCTGGTTTCCGCGG |
| *smc03872_Xba_R* | GGAAAATCTAGATCACTCCGAAATGATCTTCACCTTGTCG |
| *smc03872_Lys-_XbaI_R* | GGAAAATCTAGATTATCAGACGCGCAGCGGCTTC |
| *smc03872_His_XbaI_R* | GGAAAATCTAGATCAatggtgatggtgatggtgCTCCGAAATGATCTTCACCTTGTCG |
| *smc03872_ΔLys_His_XbaI_R* | GGAAAATCTAGATTATCAatggtgatggtgatggtgGACGCGCAGCGGCTTC |
| *smc03872*_SDMC30S_F | ACGCTGATCGCTGGCTCTCAATCCGTGATCGAG |
| *smc03872*_SDMC30S_R | CTCGATCACGGATTGAGAGCCAGCGATCAGCGT |
| *smc03872*_SDMQ29D_F | CTG ATC GCT GGC TGC GAC TCC GTG ATC GAG CAG |
| *smc03872*_SDMQ29D_R | CTGCTCGATCACGGAGTCGCAGCCAGCGATCAG |
| *smc03872*_SDMH147R_F | GAAGTGGCCGCCGTACTCTCGAGGGAGATGGCGCATGTGACGGCC |
| *smc03872*_SDMH147R_R | GGCCGTCACATGCGCCATCTCCCTCGAGAGTACGGCGGCCACTTC |
| *smc03872*_SDME148Q_F | CGTACTCTCGCACCAGATGGCGCATGTGA |
| *smc03872*_SDME148Q_R | TCACATGCGCCATCTGGTGCGAGAGTACG |
| *pkX_US_F* | CCGGCTCGTATGTTGTGTGG |
| *pkX_DS_R* | CGAAAGGGGGATGTGCTGC |
| *smc3872*_EcoRI_ F | ctgcaggaattcATGAATGGCTGGTTTCCGCGG |
| *smc3872_*HindIII_R | ctgcagaagcttTCACTCCGAAATGATCTTCACCTTGTCG |

Bold sequences = barcodes
